# Deep learning-based investigation of chloroplast translation regulatory sequences

**DOI:** 10.3389/fpls.2025.1698951

**Published:** 2025-12-11

**Authors:** Mohammad Ali Abbasi-Vineh, Pär K. Ingvarsson, Naser Farrokhi

**Affiliations:** 1 Department of Cell & Molecular Biology, Faculty of Life Sciences & Biotechnology, Shahid Beheshti University, Tehran, Iran; 2 Department of Plant Biology, Swedish University of Agricultural Sciences, Uppsala, Sweden

**Keywords:** chloroplast, Convolutional Neural Network, Shine-Dalgarno motifs, translation, algae, CNN-LSTM, genetic engineering, leader sequence

## Abstract

Understanding the architecture of translational regulatory sequences in diverse chloroplasts is critical for advancing synthetic biology and genetic engineering. In this study, a hybrid deep learning model combining convolutional neural network (CNN), long short-term memory (LSTM), Attention, and Residual architectures was developed to classify and analyse two datasets: 5′ untranslated region sequences from plants and algae, and the sequences with and without Shine-Dalgarno (SD) motifs from both groups. Using 300-nucleotide leader sequences upstream of the start codon as input, the model achieved strong prediction performance for both taxonomic origin and the presence or absence of SD motifs. However, a small subset of plant and algal sequences exhibited algal-like and plant-like patterns, respectively—an encouraging finding for identifying functional heterologous sequences from one group for use in the other group’s genome. The results further revealed significant differences in the plastid leader sequences between the datasets (Plants *vs*. Algae and SDs *vs*. without SDs), emphasising distinct features in the first 30 bp upstream of the start codon. This study proposes two potential strategies for introducing heterologous leader sequences in algal plastome engineering: (1) employing plant-derived leader sequences with algal-like patterns tailored to specific algal strains, and (2) constructing hybrid leader sequences harbouring SD motifs by fusing algae-specific ~30 bp upstream regions with their respective plant-derived distal regions. As the first deep learning model to analyse chloroplast translational regulatory sequences, the findings offer valuable guidance for identifying and predicting heterologous leader sequences in plants and algae.

## Graphical Abstract

**Figure:**
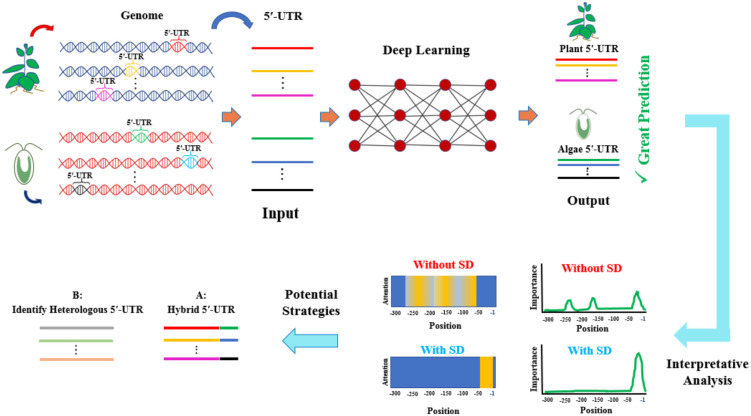
.

## 1 Introduction

Translation regulation, particularly at the initiation phase in plastids (chloroplasts) of plants and algae, represents a significant rate-limiting step for overall chloroplast gene expression (Drechsel and Bock, 2011; Trösch et al., 2018; Zoschke and Bock, 2018; Puthiyaveetil et al., 2021). This regulatory stage also limits the expression of transgenes in chloroplasts. For instance, earlier research demonstrated that the wheat *psbA* promoter, although effectively transcribed in the chloroplast of *Chlamydomonas reinhardtii*, generated transcripts that were rapidly degraded and therefore highly unstable. In addition, introducing foreign promoters and 5′ untranslated regions (UTRs) from genes such as *atpA*, *tufA*, and *psbD* led to increased accumulation of the target mRNA, but did not result in efficient translation of the transcript (Nickelsen, 1999; Gimpel and Mayfield, 2013).

The process involves a complex interplay of *cis*-elements within the 5′ untranslated regions (5′-UTRs) and their corresponding *trans*-acting protein factors, which remain incompletely understood (Odom et al., 2022; Abbasi-Vineh and Emadpour, 2024). The complexity and constraints on transgene expression during translation are significantly greater in the chloroplast of unicellular algae *Chlamydomonas reinhardtii* compared to those of *Nicotiana tabacum* plants (Kramzar et al., 2006; Gimpel and Mayfield, 2013; Abbasi-Vineh and Emadpour, 2024). These intricate interactions and negative feedback mechanisms have long hindered the identification, synthesis and utilisation of heterologous 5′-UTRs for gene expression in the *C. reinhardtii* chloroplast. To date, only the heterologous 5′-UTR from the bacteriophage T7 gene 10 (T7g10 5′-UTR) has been successfully validated for foreign protein expression in the *C. reinhardtii* chloroplast (Abbasi-Vineh and Emadpour, 2024). Despite these observations and the extensive evolutionary divergence across plants and algae, the continued conservation of chloroplast 5′-UTRs with similar structural elements remains noteworthy (Gimpel and Mayfield, 2013).

Chloroplasts retain a prokaryotic-like translation system, with 70S ribosomes and mRNAs that lack the eukaryotic 5′ caps and poly(A) tails, yet they also exhibit unique adaptations for translational regulation that distinguish them from their bacterial ancestors (Kusnetsov, 2018; Dobrogojski et al., 2020). The translation apparatus shares similarities with eubacterial systems, including reliance on Shine–Dalgarno (SD)-like sequences for translation initiation. However, not all chloroplast mRNAs conform to this mechanism. In higher plants, such as *N. tabacum, and* in microalgae like *C. reinhardtii*, some protein-coding genes lack SD-like sequences within 20 nucleotides (nt) upstream of the start codon, while others possess SD motifs at variable positions (Fargo et al., 1998; Hirose and Sugiura, 2004a; Scharff et al., 2017). This variability proposes that additional regulatory elements and factors influence translation initiation in chloroplasts. Moreover, some chloroplast and cyanobacterial mRNAs have no recognisable SD sequence within the first 200 nt upstream of their initiation codon (Hirose and Sugiura, 2004b; Kuroda et al., 2007).

This complexity necessitates the use of advanced deep learning algorithms to gain a deeper and more precise understanding of the endogenous 5’-UTR sequences of higher plants and algae. Deep learning (DL) refers to a set of machine learning algorithms based on deep neural networks (DNNs), which consist of multiple layers of artificial neural networks (Hirose and Sugiura, 2004b; Kuroda et al., 2007). Each layer contains processing units inspired by biological functions, acting as nonlinear transformation functions that process inputs in a complex manner. As the number of layers increases, the data transformation process becomes more complex, and the model’s ability to solve complicated and nonlinear problems increases dramatically. Deep learning-based methods have enabled the automatic learning of complex, multidimensional relationships from heterogeneous data across various fields, including life sciences and genetics (Mahmud et al., 2021; Mohammed et al., 2023; Chandrashekar et al., 2024; Gandhewar et al., 2025). These methods utilise various architectures such as Long Short-Term Memory (LSTM) networks and Convolutional Neural Networks (CNN) (Alzubaidi et al., 2021; Janiesch et al., 2021). Recently, hybrid CNN-LSTM models have been applied to genome sequences (Tasdelen and Sen, 2021; Kaur et al., 2022; Nandhini and Tamilpavai, 2022), marking the beginning of deep learning applications in this field.

Investigating chloroplast mRNA leader sequences using deep learning and comparative analyses across higher plants (angiosperms) and algae provides critical insights into chloroplast biology, particularly the architecture of translational regulatory elements. Such analyses may also provide strategies to overcome limitations in transgene expression in algal chloroplasts. Therefore, the present study aims to apply an optimised CNN-LSTM-Attention model with residual connections on chloroplast leader sequences across higher plants and diverse algal species to gain valuable insights into the architecture of translational regulatory elements in chloroplasts.

## 2 Results

### 2.1 CNN-LSTM-Attention-Residual model performance on the classification of leader sequences

**Figure 1:**
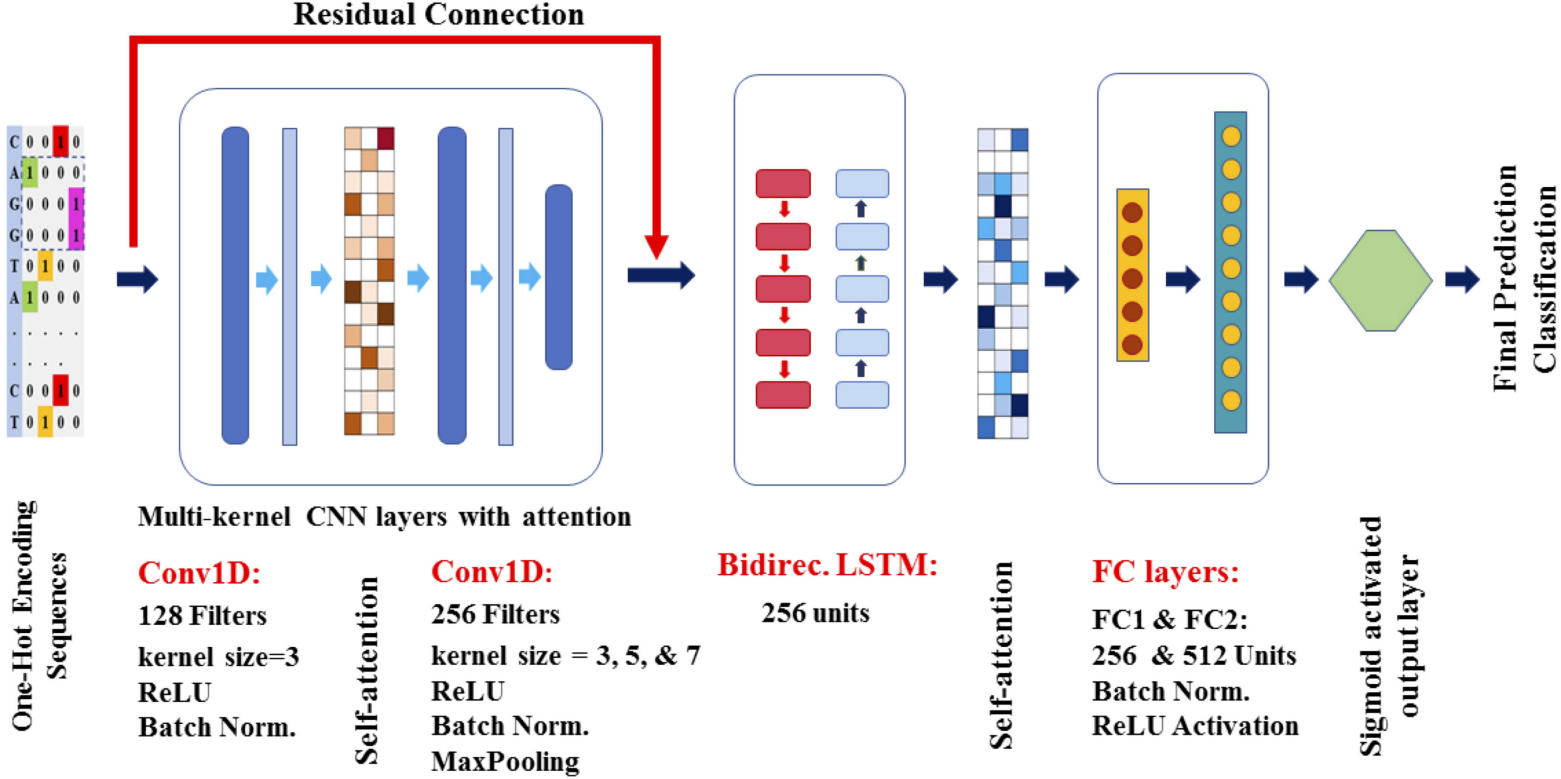
A schematic representation of the optimised CNN-LSTM model architecture with attention mechanisms was developed for leader sequence classification. This deep learning framework was applied independently to plant and algae leader sequences, as well as to sequences with and without SD motifs from these organisms. The input sequences consisted of 300-nt fragments, encoded using a four-channel one-hot representation. Outputs from the CNN and LSTM layers were further processed separately by the attention mechanism. To enhance feature propagation and mitigate potential vanishing gradient issues, a residual connection was integrated into the model. Additionally, batch normalisation was applied to each layer, and two fully connected layers were employed following the LSTM layers. Further details regarding the model architecture and implementation can be found in the Materials and Methods section.

Building upon the demonstrated efficacy of CNN-LSTM architectures in genomic sequence analysis (Abbasi-Vineh et al., 2025), an enhanced hybrid model incorporating attention mechanisms was implemented for the classification of leader sequences (**Figure 1**). This optimised architecture utilised CNN layers with multiple kernel sizes combined with Bi-LSTM layers. To improve sequence modelling, feature normalisation was scaled for each layer. Meanwhile, the attention layers dynamically weighted critical regions of the sequences identified through the deep operations of both networks, enabling more precise recognition of regulatory motifs.

**Figure 2:**
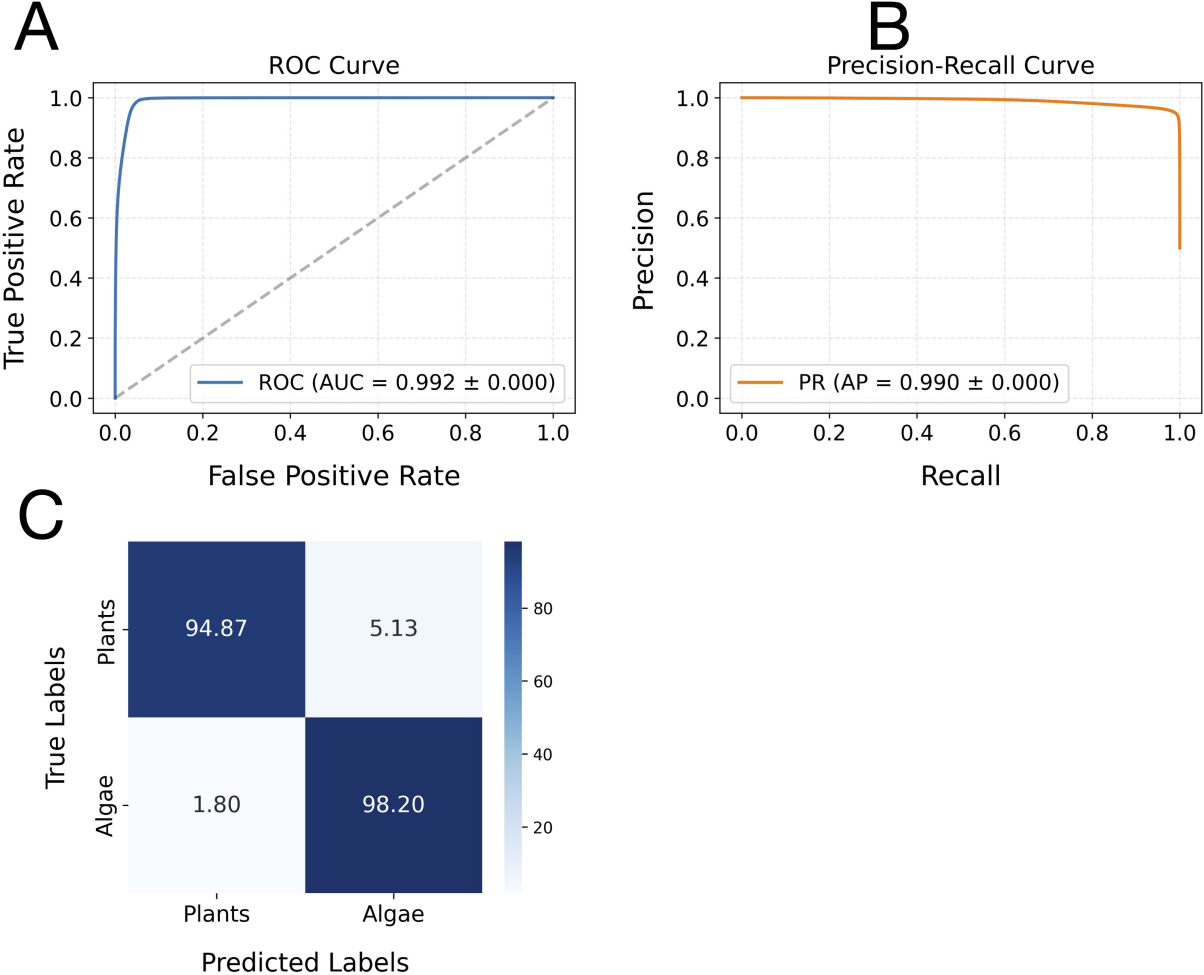
Receiver operating characteristic (ROC) and precision-recall (PR) curves, along with the confusion matrix analysis, for the classification model on plant and algae leader sequences. Panel **(A)** displays the ROC curve, where the x-axis represents the false positive rate and the y-axis indicates the true positive rate. Panel **(B)** shows the PR curve, with recall (sensitivity) on the x-axis and precision on the y-axis. Both curves were generated from the model’s predictions on the leader sequence datasets. The average area under the ROC curve (AUC) and average precision (AP), calculated across multiple runs, are annotated on each plot to summarise overall model performance. Panel **(C)** presents the confusion matrix analysis of the CNN-LSTM model’s classification performance. The matrix displays binary classification results, with rows corresponding to the actual class labels (Algae and Plants) and columns indicating the predicted class labels. Diagonal elements (top-left for Plants, bottom-right for Algae) represent the proportions of correctly classified samples. In contrast, off-diagonal elements correspond to the percentages of misclassified leader sequences between plants and algae.

The hybrid CNN-LSTM model demonstrated exceptional, consistent performance in distinguishing leader sequences between plant and algal groups, as evaluated via 5-fold cross-validation. Across all folds, the model achieved an accuracy of 0.96 ± 0.02, with identical stability in weighted F1-score (0.96 ± 0.00) and Matthews correlation coefficient (MCC: 0.93 ± 0.01), indicating perfect reproducibility in classification performance. Both macro- and weighted-average metrics (precision: 0.96 ± 0.01; recall/F1-score: 0.96 ± 0.00) confirmed that the model maintained balanced precision and sensitivity across classes without inter-fold variability. The classification model’s performance was further evaluated using both the receiver operating characteristic (ROC) and precision-recall (PR) curves (**Figure 2**). The ROC curve demonstrated a high true-positive rate across a broad range of false-positive rates, with an area under the curve (AUC) of 0.992 ± 0.00 (**Figure 2A**), indicating excellent discriminative ability. Similarly, the PR curve showed consistently high precision over a wide range of recall values, with an average precision (AP) of 0.99 ± 0.00 (**Figure 2B**). These results collectively suggested that the model achieved outstanding classification performance, maintaining both high sensitivity and precision across various threshold settings.

The confusion matrix further elucidated the CNN-LSTM model’s exceptional performance in discriminating between plant and algae leader sequences. The model correctly predicted 98.20% of algae sequences (true positives) and misclassified only 1.80% as plant sequences (false negatives). Conversely, 94.87% of plant sequences were accurately identified, while 5.13% were misclassified as algae (**Figure 2C**). This asymmetry shows that algal leader sequences contain more distinctive diagnostic features.

Notably, a small subset of plant sequences (5.13%) exhibited algal-like patterns (**Figure 2C**), suggesting that these sequences could be candidate heterologous leader sequences for algal plastomes.

### 2.2 Differential attention patterns for leader sequences in algae and plants

**Figure 3:**
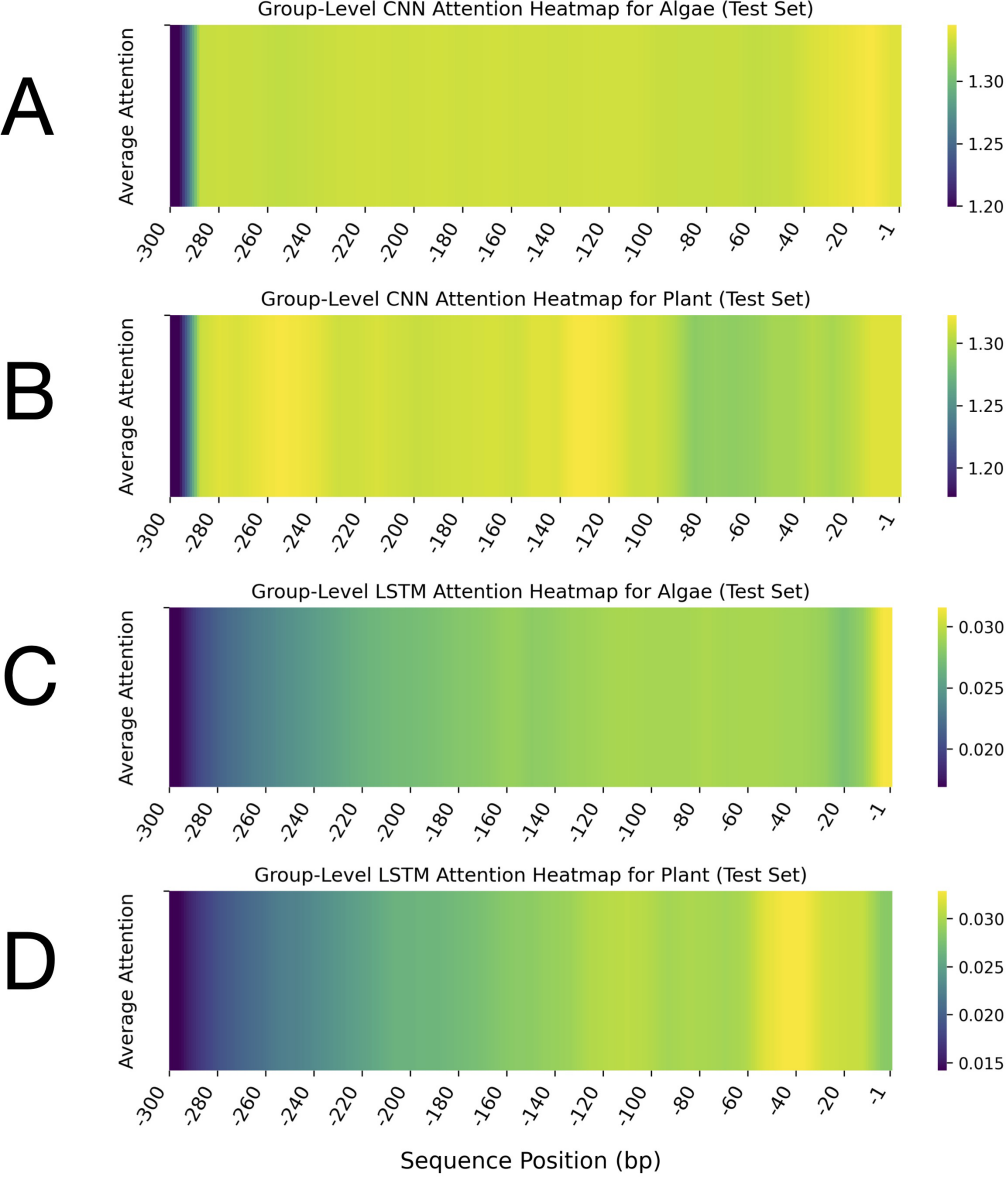
Group-level attention heatmaps for leader sequences from algae and plant groups, computed by CNN and LSTM models on the test set. These heatmaps depict the average attention scores assigned to each nucleotide position within the leader sequences, highlighting the sequence regions prioritised by each model during classification. Panels **(A)** (algae) and **(B)** (plants) show CNN attention heatmaps generated by the model for leader sequences. Panels **(C)** (algae) and **(D)** (plants) display LSTM attention heatmaps produced by the model for leader sequences. In all plots, the x-axis represents the sequence position in base pairs (bp), ranging from -1 to -300 upstream of the translation start site. The y-axis denotes the average attention score. Each heatmap includes a vertical colour bar on the right that indicates the mapping between colour intensity and average attention values. Note that the colour scale ranges differ between the CNN and LSTM heatmaps. Across all panels, darker colours (purple/blue) correspond to lower attention scores, whereas lighter colours (yellow/green) indicate higher attention.

The comparative analysis of leader sequences between algae and plants, based on CNN and LSTM attention heatmaps, revealed distinctive patterns in sequence importance and regulatory architecture (**Figure 3**). The results of CNN-based attention analysis, which emphasised spatial features, revealed prominent differences between the leader sequences of the two groups (**Figures 3A, B**). For the algal sequences, the CNN attention heatmap displayed remarkably high and consistent attention across nearly the entire leader sequence, with only reduced attention at the 5’ end (-285 to -300 bp) (**Figure 3A**). This uniform pattern suggested that the entire 285-nt length of the leader sequences may contribute to the regulatory functions. In contrast, the plant leader sequences analysed by CNN displayed a more heterogeneous attention pattern with distinct peaks and valleys. Several regions showed heightened attention, interspersed with regions of moderate attention (**Figure 3B**). This suggests that plant leader sequences may contain multiple discrete regulatory elements distributed across specific regions, rather than relying on a uniform composition.

LSTM-derived heatmaps, which emphasise capturing long-range dependencies, showed a gradual increase in attention from the 5’ end toward the 3’ end (position -1) of the algal sequences, reaching maximum intensity within the first 10 bp upstream of the start codon (-1 to -10 bp) (**Figure 3C**). This gradient was marginally steeper in plant sequences compared to algae. The LSTM-derived attention heatmaps for plant sequences showed a clear gradient, with attention values increasing progressively from distal regions (-300 bp) to proximal regions (-1 bp) of the 5’-UTR. The highest attention values were concentrated within the -10 to -60 bp regions upstream of the start codon of the plant sequences (**Figure 3D**). The LSTM attention heatmap also revealed a heterogeneous attention pattern within the -1 to -160 bp region, which was not observed in the corresponding region of the algal sequences.

These results indicate that distinct regions with sequence-specific features and varying attention patterns within leader sequences contribute to the differentiation between algal and plant leader sequences, despite their shared cyanobacterial ancestry.

### 2.3 Group-level saliency and perturbation test analyses of leader sequences in algae and plants

**Figure 4:**
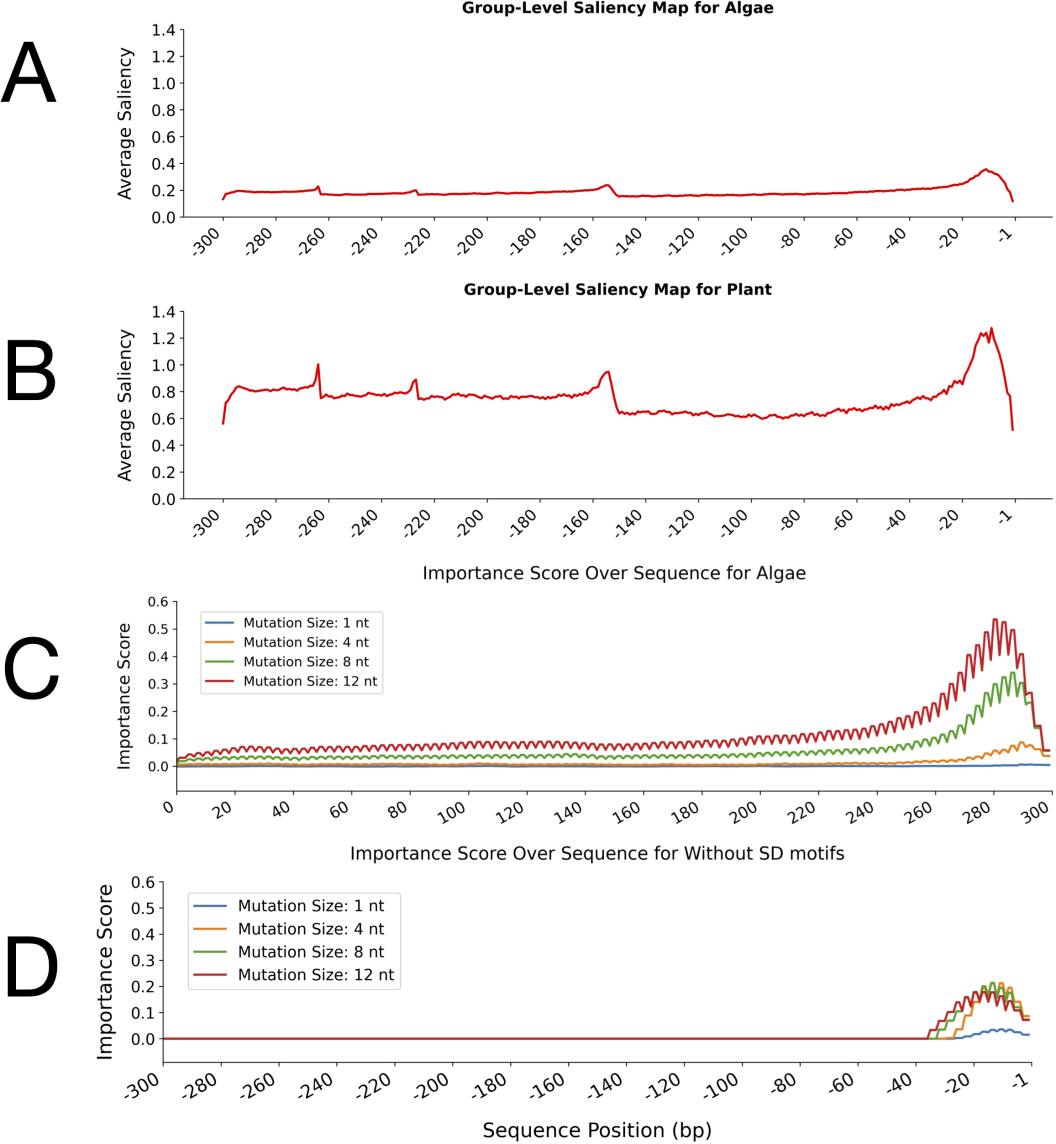
Group-level saliency maps and perturbation test importance scores for leader sequences in algae and plant groups. **(A, B)** Average saliency values across nucleotide positions for algae **(A)** and plant **(B)** leader sequences, as learned by the CNN-LSTM model. The x-axis indicates sequence position (bp) upstream of the start codon, and the y-axis represents the average saliency score. The average saliency score at each nucleotide position reflects the relative importance of that position for classification as determined by the model. **(C, D)** Importance scores from perturbation tests with different mutation sizes (1, 4, 8, and 12 nucleotides) along the sequence in algae **(C)** and plants **(D)**, generated using the hybrid model. The x-axis shows sequence position (bp) upstream of the translation start codon, and the y-axis represents the average importance score.

The group-level saliency maps of the leader sequences for algal and plant groups further emphasised the differences between their sequences and highlighted the corresponding important regions (**Figures 4A, B**). The saliency maps indicate the incidences of the sequences to which the model assigns the most attention. In the algae group, the average saliency values remained low across most of the sequences with only minor fluctuations and a modest increase toward the 3′ end (positions -1 to -20 bp) (**Figure 4A**). However, the plant group showed higher overall baseline saliency across the leader sequences than the algae group. In the plant saliency map, several distinct peaks were observed, with the most pronounced increase occurring near the translation start site (positions -1 to -20 bp) (**Figure 4B**).

The perturbation tests further validated the distinct patterns observed between the leader sequences by introducing mutations of varying sizes (1, 4, 8, and 12 nt) to assess the robustness of the identified important regions (**Figures 4C, D**). These tests revealed where mutations exert the greatest functional impact on the model’s predictions. In algal sequences, importance scores increased sharply toward the 3′ end, with larger mutations (8 nt and 12 nt) producing higher scores, reaching up to 0.5 for 12-nt mutations. Even single-nucleotide mutations caused measurable effects near the 3′ end, indicating that this region is particularly sensitive to sequence alterations (**Figure 4C**). In contrast, perturbation tests on plant sequences showed that mutations predominantly increased importance scores within the 3′ end of the leader sequences, specifically between nucleotides -1 and -30 relative to the start codon (**Figure 4D**). However, despite this localised increase, overall importance scores in plant sequences remained consistently lower than those observed in algae.

The results of these analyses revealed distinct regulatory patterns between algae and plants. In plants, higher baseline saliency across many positions suggested widespread relevance. Still, lower importance scores indicated that no single region is critical, reflecting a more distributed and mutation-robust regulatory logic, possibly due to compensatory elements. In contrast, algae exhibited lower overall saliency but much higher importance scores concentrated at the 3′ end, highlighting a focussed and essential regulatory region. Perturbation tests revealed that mutations, particularly 8- and 12-nt changes, increased importance scores throughout the algal leader sequences, with the strongest effects observed at the 3′ end. For plants, mutations only increased importance scores near the 3′ end. These findings indicated that the 3′ end contains key regulatory elements indispensable for model predictions in both groups, but this region was more critical for algae. Together, these complementary analyses provided a comprehensive understanding of sequence-function relationships as captured by deep learning models.

### 2.4 Differentiation of leader sequences with and without SD motifs in algae and plants

The same hybrid CNN-LSTM model used in the previous sections was also separately applied to analyse sequences with or without Shine-Dalgarno (SD) motifs located 20 nucleotides upstream of the start codon in both algae and plants. For algae, the dataset comprised 73,159 sequences without SD motifs and 37,258 sequences with SD motifs. In plants, there were 372,992 sequences without SD motifs and 511,045 sequences with SD motifs. Balanced subsets were constructed for model evaluation, resulting in 74,516 algae sequences (37,258 with and 37,258 without SD motifs) and 400,000 plant sequences (200,000 with and 200,000 without SD motifs).

**Figure 5:**
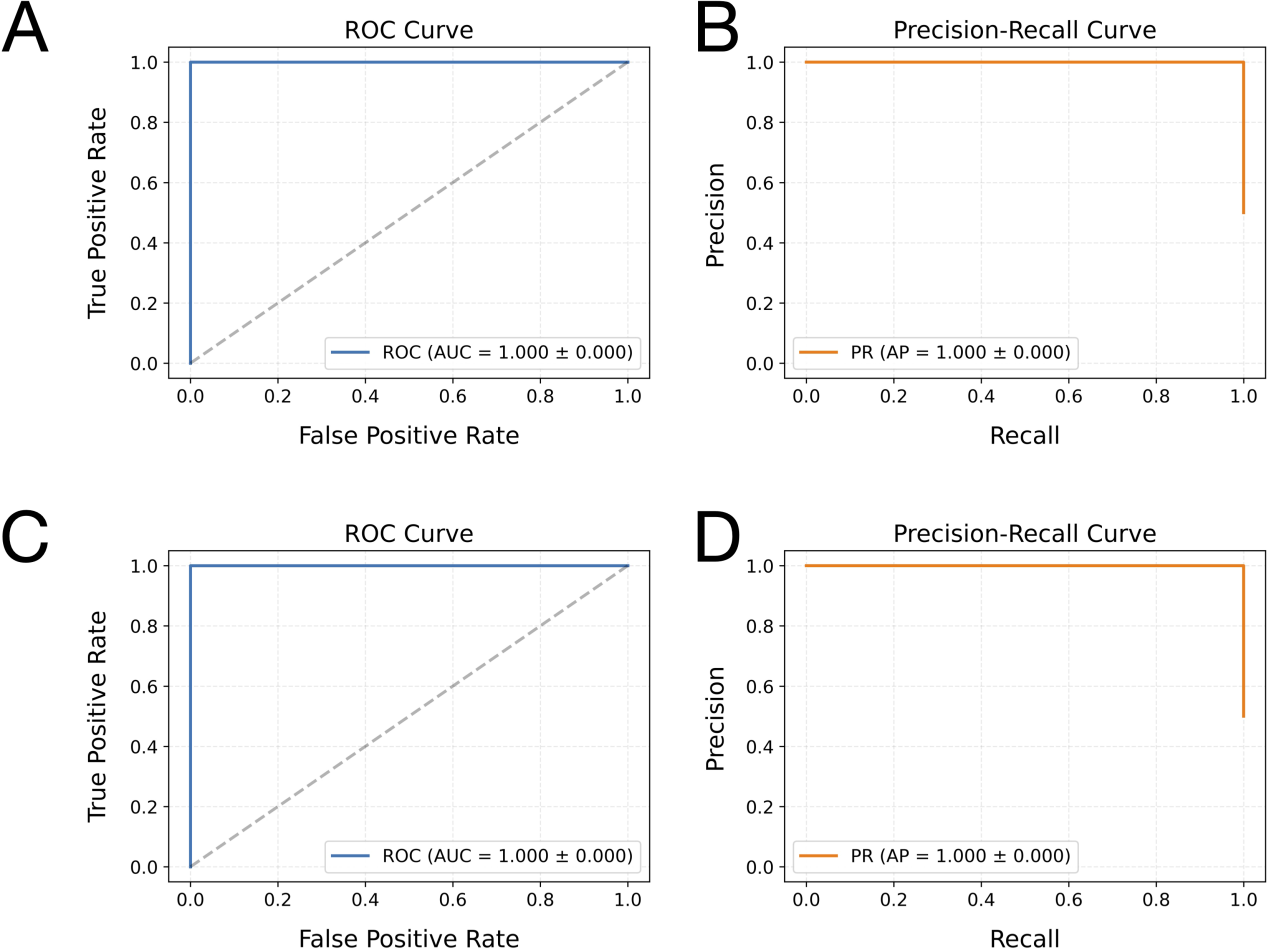
Receiver operating characteristic (ROC) and precision-recall (PR) curves illustrating the classification model’s performance on leader sequences with and without SD motifs in both plant and algal groups. Panels **(A, C)** present the ROC curves for algal and plant sequences, respectively. Panels **(B, D)** display the PR curves for algal and plant sequences, respectively. These curves are based on the model’s predictions using the sequence test dataset. Each plot includes the average area under the ROC curve (AUC) and average precision (AP) values, computed across multiple runs, to provide a summary of the model’s overall accuracy.

The CNN-LSTM models demonstrated near-perfect discrimination between leader sequences with and without SD motifs in both algae and plant datasets. For algae, the model achieved an accuracy of 0.99 ± 0.02, a weighted F1 score of 0.99 ± 0.01, and an MCC of 0.99 ± 0.01. Precision-recall and ROC AUC values were both 1.00 ± 0.00 (**Figures 5A, B**), indicating exceptional classification performance. Similarly, for plants, the model achieved an accuracy of 0.99 ± 0.00, a weighted F1 score of 0.99 ± 0.00, and an MCC of 0.99 ± 0.03, with both precision-recall and ROC AUC values of 1.00 ± 0.00 (**Figures 5C, D**).

The confusion matrices for both groups further confirmed the outstanding performance, with virtually all sequences correctly classified into their respective categories (**Supplementary Figure S1**). These results highlighted the distinct sequence features associated with the presence or absence of SD motifs in the leader sequences of both algae and plants. They underscored the effectiveness of the CNN-LSTM model for this classification task.

Interestingly, although a low number of leader sequences containing SD motifs showed patterns comparable to those lacking SD motifs, none of the leader sequences without SD motifs displayed characteristics similar to those with SD motifs (**Supplementary Figure S1**).

### 2.5 Comparative analysis of attention heatmaps for algal and plant leader sequences with and without SD motifs

**Figure 6:**
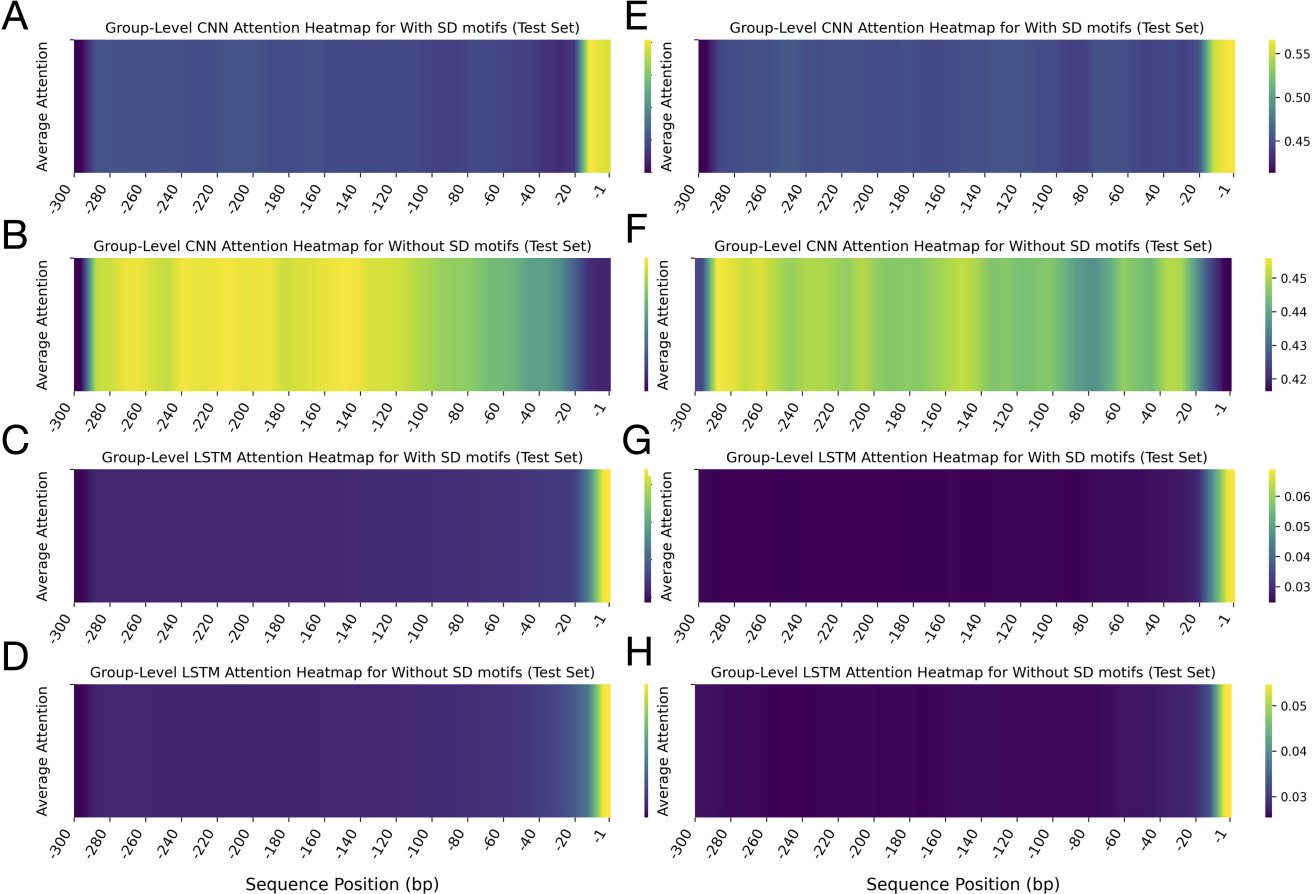
Group-level attention heatmaps for algae and plant leader sequences with and without Shine-Dalgarno (SD) motifs. The heatmaps show average attention scores calculated by CNN and LSTM models. The x-axis marks sequence positions (bp) from -1 to -300 upstream of the translation start site. The top four panels display results for algae: **(A, B)** CNN attention heatmaps for sequences with and without SD motifs, respectively; **(C, D)** LSTM attention heatmaps for sequences with and without SD motifs, respectively. The y-axis represents average attention scores. The bottom four panels show corresponding results for plant sequences: **(E, F)** CNN attention heatmaps with and without SD motifs, and **(G, H)** LSTM attention heatmaps with and without SD motifs. Each heatmap includes a vertical colour bar on the right side, which shows the correspondence between colour intensity and average attention values. Note that the colour scales differ in range between the heatmaps. Across all panels, darker shades (purple/blue) indicate lower attention scores, while lighter shades (yellow/green) indicate higher attention.

Analysis of group-level attention heatmaps generated by the CNN and LSTM models revealed distinctive patterns across algal leader sequences with and without SD motifs (**Figure 6**; top four panels). The heatmaps showed striking differences in CNN attention distributions between sequences with and without SD motifs in algae (**Figures 6A, B**). The attention heatmaps exhibited predominantly low attention across most of the sequence length, with a dramatic shift to high attention at the 3’ terminal region (positions -1 to -20) in algae sequences. This sharp contrast indicated that when SD motifs were present, the CNN model strongly prioritised the downstream region of the leader sequences, where these motifs typically occurred. In contrast, for sequences lacking SD motifs, the CNN model distributed its attention more broadly across the upstream region, with elevated attention spanning a wider range of positions and peaking further upstream from the start codon (at the position between ∼ -120 to -290) (**Figures 6A, B**). Interestingly, the 3’ terminal region, which received high attention in SD-containing sequences, displayed comparatively lower attention here. This proposed that, in the absence of canonical SD motifs, the CNN model may rely on broader contextual cues within the leader sequences to inform its predictions. The LSTM model, on the other hand, demonstrated a more uniform attention profile across most positions of the algae and plant sequences, with heightened focus at the 3’ end (-1 to -15) for both SD-containing and SD-lacking sequences (**Figures 6C, D**).

Collectively, these results indicate that the CNN model was more adept at focussing on SD motifs when they were present, whereas when they were absent, the model adopted a broader scanning strategy across the sequence. In contrast, the LSTM model consistently allocated the highest attention to the 3′ end of the leader sequences in both groups, regardless of whether the SD motif was present or absent.

Analysis of the group-level attention heatmaps for plant leader sequences, stratified by the presence or absence of SD motifs, revealed behaviours similar to those of algae (**Figure 6**; bottom four panels). In the CNN-derived heatmaps, sequences containing SD motifs showed predominantly low attention values across most of the leader sequences, with a pronounced increase in attention localised at the 3′ terminal region (positions -1 to -20) (**Figure 6E**). In contrast, for sequences lacking SD motifs, the CNN attention was more broadly distributed, with higher values observed at the 5′ end and in several central regions. In comparison, the 3′ end received comparatively less attention (**Figure 6F**). This also showed that, in the absence of SD motifs, the model shifted its focus to regions that may harbour alternative regulatory elements for SD motifs. On the other hand, the LSTM attention heatmaps for the plant sequences displayed a consistent trend regardless of SD motif presence, with the highest attention values at the 3′ end (positions -1 to -20) (**Figures 6G, H**).

Overall, both CNN and LSTM models highlighted the functional importance of the 20 nucleotides upstream of the start codon in algae and plant leader sequences, particularly when SD motifs were present. These attention patterns align with the known biological role of SD motifs in translation initiation, suggesting that the deep learning model is sensitive to the presence of key regulatory elements in algal and plant leader sequences.

### 2.6 Saliency map and perturbation test analyses for algal and plant leader sequences with and without SD motifs

**Figure 7:**
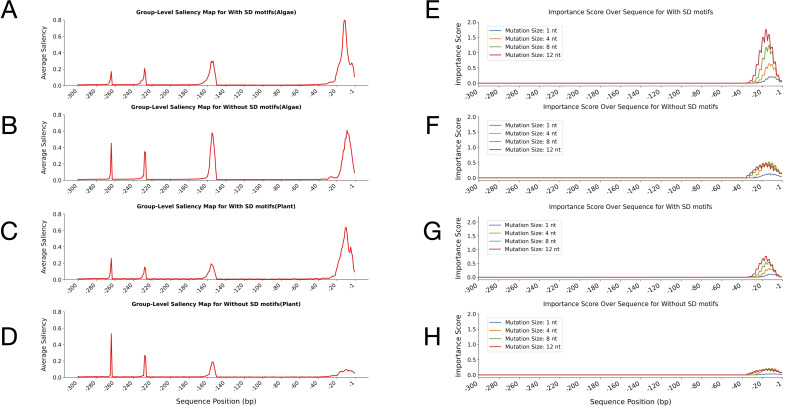
Group-level saliency maps and perturbation-test importance scores across leader sequences from algae and plants, with and without Shine-Dalgarno (SD) motifs. The top four panels display saliency maps, showing average saliency values plotted across sequence positions relative to the start codon. Panels **(A, B)** correspond to algae sequences with and without SD motifs, respectively, while panels **(C, D)** correspond to plant sequences with and without SD motifs, respectively. In all saliency map visualisations, the x-axis represents sequence positions in base pairs (bp), ranging from -1 to -300 upstream of the translation start site, and the y-axis shows the average attention score at each position. The bottom four panels show the perturbation-test importance scores for the corresponding algae and plant sequences, calculated using a CNN-LSTM model. Importance scores are reported for different mutation sizes (1, 4, 8, and 12 nucleotides) along the leader sequences. Panels **(E, F)** show the perturbation test results for algae sequences with and without SD motifs, respectively. In contrast, panels **(G, H)** show results for plant sequences with and without SD motifs, respectively. For the perturbation tests, the x-axis indicates sequence positions, and the y-axis represents the average importance score.

Group-level saliency map analysis further confirmed the distinct model attention patterns observed in previous heatmap analyses for both algal and plant leader sequences with and without SD motifs (**Figure 7**; top four panels). In both algae and plants, the sequences containing SD motifs exhibited a pronounced and sharply localised peak in saliency at the 3′ terminal region (positions approximately -1 to -20) (**Figures 7A, C**). This indicated that, when SD motifs were present, the model’s predictive focus was almost exclusively on the region immediately upstream of the start codon, consistent with the canonical location of SD motifs. Additional minor peaks were observed upstream (around -150, -220, and -260), although these were less pronounced (**Figures 7A, C**).

In contrast, sequences lacking SD motifs in algae sequences displayed a broader saliency distribution, with prominent peaks shifted further upstream (around -150, -220 and -260), and also a modest increase at the 3′ end (positions -1 to -20) (**Figure 7B**). This redistribution of model focus in the absence of SD motifs suggested that the model relied on alternative sequence features or regulatory signals located in upstream regions. For plant sequences lacking SD motifs, the saliency map again showed a redistribution of model focus. The most prominent peaks shifted upstream (around -260, -220, and -150), with only the lowest elevation in saliency at the 3′ end (positions -1 to -20) (**Figure 7D**). This clearly mirrors the pattern observed in the algae sequences and reinforces the model’s adaptive strategy when SD motifs are absent. Additionally, it confirmed that attention was lower in plant leader sequences lacking SD motifs than in those containing SD motifs (**Figures 6G, H**).

To further validate the distinct positional importance of SD motifs within the leader sequences of algae and plants, a systematic mutational importance analysis was performed on sequences with and without SD motifs from both groups, separately (**Figure 7**; bottom four panels). For both algae and plant datasets, the importance score profiles consistently revealed that, regardless of mutation size, sequences containing SD motifs exhibited sharply elevated importance scores at the extreme 3′ end of the leader sequences (proximal to the start codon, positions ~ -1 to -30 bp) (**Figures 7E, G**). This pattern was robust across all tested mutation window sizes (1, 4, 8, and 12 nt), indicating that the downstream region containing the SD motifs was critical for model predictions.

On the other hand, sequences lacking SD motifs still showed a concentration toward the 3′ end, but with lower peak scores than those containing SD motifs (**Figures 7F, H**). Furthermore, the peak importance scores in algae with and without the SD motifs appeared slightly higher than those in plants, suggesting a potentially stronger or more specific reliance on the terminal regions in algae.

### 2.7 Sequence logo analysis for leader sequences in algae and plants

**Figure 8:**
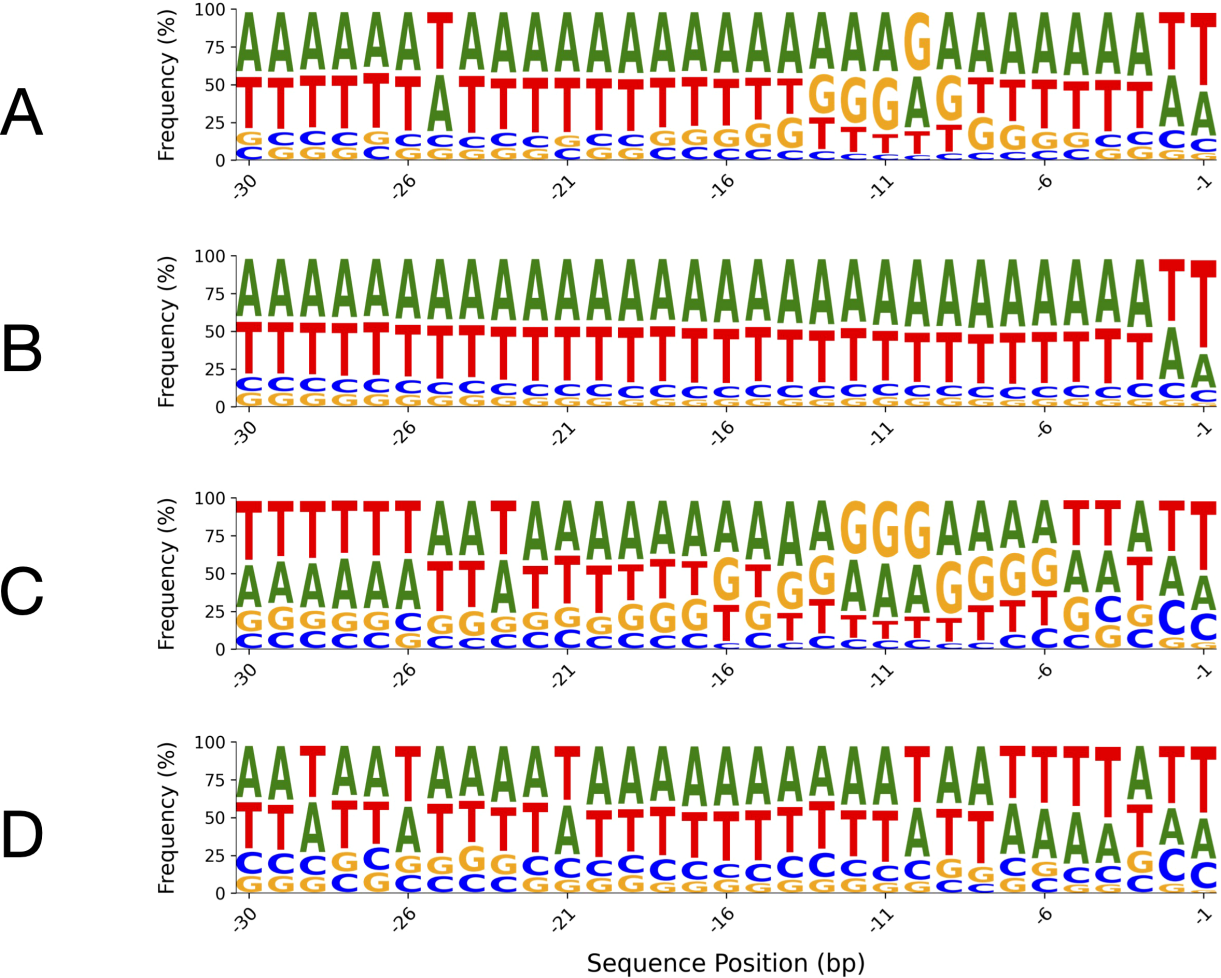
Sequence logos of leader sequence regions (positions -1 to -30 bp relative to the start codon) in algae and plants, with and without SD motifs. The top panels show algal 5′-UTRs: **(A)** with SD motifs and **(B)** without SD motifs. The bottom panels show plant 5′-UTRs: **(C)** with SD motifs and **(D)** without SD motifs. The canonical Shine–Dalgarno sequence in chloroplast genomes is generally “AGGAGG” or closely related variants, positioned upstream of the start codon. For sequence logos generated over the entire 300-nucleotide length, refer to **Supplementary Figures S2**, **S3** in the **Supplementary File**.

Sequence logos were generated for the -1 to -30 bp region upstream of the start codon in both algal and plant leader sequences, comparing sequences with and without SD motifs (**Figure 8**). In sequences lacking SD motifs (**Figures 8B, D**), both algae and plants exhibited a strong enrichment of adenine (A) and thymine (T), resulting in a pronounced AT-rich profile across the region. This AT-richness was exceptionally uniform in algal sequences (**Figure 8B**) compared to plant sequences (**Figure 8D**). In contrast, sequences containing SD motifs (**Figures 8A, C**) showed a notable increase in guanine frequency, particularly at positions corresponding to the canonical SD site, with some elevation in cytosine as well. This shift was more pronounced in plant sequences with SD motifs (**Figure 8C**), which showed a higher frequency of guanine-containing sites than their algal counterparts (**Figure 8A**). Additionally, the uniformity of A and T frequencies remained higher in algal sequences than in plant sequences, regardless of SD motif presence.

Sequence logos generated over the entire 300-nucleotide length revealed that the high abundance of AT-rich sequences is a common characteristic shared by both algal and plant leader sequences (**Supplementary Figures S2**, **S3**). Notably, the sequence logos for algae exhibited greater uniformity in this AT-rich region than those for plants. A prominent feature observed throughout these sequences is the presence of oligo-A and oligo-T tracts of varying lengths (**Supplementary Figures S2**, **S3**). Analysis of their frequency identified motifs such as “AAAAAA,” “TTTTTT,” “AAAAAT,” and “TAAAAA” as among the most abundant in algal and plant leader sequences (**Supplementary Figure S4**). Importantly, these motifs showed higher prevalence in the -1 to -30 bp region of leader sequences containing SD motifs compared to those lacking SD elements (**Supplementary Figures S5**, **S6)**. Further examination of 6-mer motif abundance within this region indicated that, in leader sequences lacking SD motifs, the frequency of these oligo-A/T motifs increased (**Supplementary Figure S7**). This suggests a potential compensatory role for these motifs in ribosomal subunit binding where the canonical SD sequence is absent. However, given that these motifs were found in both SD-containing and SD-lacking sequences, albeit at different frequencies, a strictly unique functional equivalence to the SD motif cannot be definitively ascribed.

### 2.8 Potential heterologous leader sequences for algal plastome engineering

Based on the results obtained from the present study, two strategies are proposed for utilising heterologous sequences of chloroplast origin with appropriate efficiency in algae: (A) the use of plant-driven heterologous sequences with algal-like patterns, and (B) the development of hybrid leader sequences.

#### 2.8.1 Use of heterologous sequences with algal-like patterns

The results from the CNN-LSTM model revealed that 5.13% of plant sequences were misclassified as algal sequences due to their high similarity to their patterns (**Figure 2C**). Among the 110,417 plant sequences examined, 5,399 sequences exhibited patterns similar to algal sequences; these sequences are provided in **Supplementary Data S1**. Analysis confirmed that none of these plant sequences matched any algal sequences, indicating they were unique heterologous candidates. Among these sequences, 2,151 contained the canonical SD sequences within the first 30 nucleotides upstream of the genes (-1 to -30 of leader sequences), while 3,248 sequences lacked SD sequences in this region. This suggests that these plant-origin heterologous sequences represent promising candidates for further investigation in algal plastome applications. Conversely, 1,556 algal sequences were misclassified as plant sequences by the model (**Supplementary Data S2**).

#### 2.8.2 Development of hybrid leader sequences

The results demonstrated that the 30-nt region at the 3’ termini of leader sequences (positions -1 to -30) constituted a critical and influential region for accurate classification of plant and algal sequences. When this region was removed from the sequences, the model’s performance on partial sequences (270 nt, spanning positions -30 to -300) was substantially reduced compared to full-length sequences (300 nt). The truncated dataset yielded weighted precision, recall, and F1-scores of 0.712 ± 0.02, 0.512 ± 0.01, and 0.365 ± 0.02, respectively, demonstrating significantly impaired classification performance. For further evaluation, refer to the results of the ROC and PR curves for this dataset in **Supplementary Figures S8A, B**. These results confirmed the sequence-specific nature of the -1 to -30 bp region for each taxonomic group.

The confusion matrix analysis revealed that while the model correctly predicted 99.97% of algae sequences using partial sequences, approximately 99.63% of plant sequences were misclassified as algal sequences (**Supplementary Figure S8C**). This pattern indicated that all truncated plant sequences exhibited algal-like characteristics when the terminal 30 nucleotides were removed. Consequently, the construction of hybrid sequences incorporating partial plant-origin heterologous sequences combined with algae-specific -1 to -30 regions represents a viable strategy for optimising expression in algal plastomes.

Overall, **Table 1** presents a summarised comparison of the performance metrics of the CNN-LSTM-Attention-Residual model applied to algae and plant leader sequences, using both the full length of 300 nt and partial leader sequences of 270 nt. The table also separately displays the model outputs for each dataset. This provides a clear comparative overview of their classification efficacy and the model’s potential for future practical applications.

**Table 1:** Comparison of performance metrics and outcomes of the CNN-LSTM-Attention-Residual model on leader sequences of algae and plants.

For leader sequences with the full length of 300 nt (positions -1 to -300)							
		Evaluation metrics			Number of misclassified sequences as outputs		
	Number of inputs	Precision	Recall	F1-score	Total	With SD	Without SD
Algae	110,417	0.950	0.982	0.965	1,556	944	613
Plants	110,417	0.981	0.948	0.964	5,399	2,151	3,248
For partial leader sequences of 270 nt, spanning positions -30 to -300							
		Evaluation metrics			Number of misclassified sequences as outputs		
	Number of inputs	Precision	Recall	F1-score	Total	With SD	Without SD
Algae	110,417	0.506	0.999	0.667	110,008	72,959	37,049
Plants	110,417	0.931	0.004	0.007	35	15	20

## 3 Discussion

Plants and algae are evolutionarily distinct groups with different structural characteristics. They occupy contrasting environments: plants primarily inhabit terrestrial habitats, whereas algae are predominantly found in aquatic environments. Despite having evolved independently for several hundred million years, plants and algae maintain highly conserved chloroplast structures and photosynthetic functions, along with numerous chloroplast 5′-UTRs that share similar structural features within this regulatory region (Gimpel and Mayfield, 2013). Although plastid coding sequences and promoters can be functionally exchanged even between distantly related species, the functional compatibility of 5′-UTRs appears to be restricted to closely related organisms, a deduction made based on a small subset of 5′-UTRs (Nickelsen, 1999; Kramzar et al., 2006; Gimpel and Mayfield, 2013; Abbasi-Vineh and Emadpour, 2024). This underscores the importance of precisely characterising leader sequence variation, particularly when employing heterologous 5′-UTRs in the algal plastome, such as that of *C. reinhardtii*. Consequently, the application of our current advanced deep learning approach has been essential for analysing the similarities and differences of leader sequences between plants and algae.

The results demonstrated that the optimised CNN-LSTM-Attention-Residual architecture achieved high, consistent performance, with evaluation metrics approaching perfection across all tested datasets and cross-validation folds of leader sequence data. This robust performance was achieved despite the leader sequences sharing similar characteristics, including approximately 70% AT content and numerous stretches containing multiple adenines, thymines, or combinations thereof. These results were in agreement with previous studies highlighting the efficacy of hybrid CNN-LSTM models for genomic sequence analysis, particularly for plastome sequences (Kaur et al., 2022; Nandhini and Tamilpavai, 2022; Abbasi-Vineh et al., 2025). The integration of attention mechanisms and residual connections further enhanced the model’s ability to dynamically prioritise critical regulatory motifs, as supported by recent advances in deep learning for biological sequence analysis (Liu and Gong, 2019; Shen et al., 2021; Tasdelen and Sen, 2021; Pan et al., 2023). Supporting the biological interpretability of these components, the attention-derived positional signals identified in this study could be directly associated with known translational elements such as the Shine–Dalgarno (SD) motif, conserved poly(U)-rich regions, and RNA secondary structure domains involved in ribosome recruitment and stabilisation. Established sub-chloroplast localisation studies, such as PredAlgo (Tardif et al., 2012) and more recent DL-based protein sub-chloroplast localisation and mRNA subcellular localisation frameworks Kong et al., 2024a, 2024b, 2024c), further reinforce this interpretation by illustrating how deep learning models can recognise conserved spatial–regulatory patterns within regulatory regions.

Beyond model performance and despite their overall conservation (Green, 2011; Dobrogojski et al., 2020), the results revealed notable differences between plastid leader sequences of algae and plants. Evolutionary divergence appears to confer specific regulatory characteristics on these sequences. This was supported by a confusion matrix analysis, which showed that 94.87% of plant sequences and 98.20% of algal sequences possess unique, group-specific regulatory features despite their shared ancestral genome and bacterial-type regulatory systems (Drechsel and Bock, 2011; Zoschke and Bock, 2018; Dobrogojski et al., 2020). However, 5.13% of plant leader sequences (5,399 sequences) exhibited patterns similar to those of algae, and 1.80% of algal sequences (1,556 sequences) displayed features resembling plant sequences—an encouraging finding for genetic engineering and plastid synthetic biology. These plant- and algae-derived sequences represent promising candidates for further study and potential use in designing plastid expression systems tailored to the genomes of algal and plant species, respectively. This is particularly advantageous in algal biotechnology, where transgene expression in the plastome is more confined to endogenous translational regulatory elements. Introducing heterologous leader sequences into algal chloroplasts can help minimise unwanted homologous recombination and reduce adverse regulatory effects (Rasala et al., 2011; Abbasi-Vineh and Emadpour, 2024).

Thus, one promising strategy emerging from the current study is that the dataset of plant-origin heterologous leader sequences (available in **Supplementary Material S2**) could have significant potential for application across diverse algal genera and species, benefiting both research and commercial strains. The significance of this finding is underscored by the fact that only one single bacteriophage-derived leader sequence (T7g10 5′-UTR) has been effectively demonstrated to drive heterologous protein expression in the *C. reinhardtii* chloroplast (Abbasi-Vineh and Emadpour, 2024). Since a maximum length of plastid leader sequences was considered in this study (300 nt), these sequences could even include promoter regions. This could be useful for designing algal chloroplast constructs because, in addition to selecting an appropriate 5′-UTR, designing the chimeric promoter and 5′-UTR structure also affects translation efficiency (Rasala et al., 2011; Gimpel and Mayfield, 2013), likely contributing to both transcript stability and translation initiation. Further bioinformatic analyses of sequence secondary structure—such as evaluating GC content and minimum free energy (MFE) of SD-containing regions within the target host genome—could provide valuable insights for identifying optimal leader candidates. For example, the GC content of 300-nt *C. reinhardtii* leader sequences containing SD motifs ranges from 18.3% to 38%, with an average of 27.06%. In contrast, their corresponding MFE values range from −38.9 to −92.8 kcal/mol, averaging −58.46 kcal/mol (calculated using the RNAfold web server: http://rna.tbi.univie.ac.at/cgi-bin/RNAWebSuite/RNAfold.cgi). Transferring deep learning models or developing RNA structure-based models could also be valuable in advancing this issue (Foley et al., 2015; Komatsu et al., 2020; Sato et al., 2021; Fu et al., 2022).

The comparative attention heatmap analysis confirmed distinct differences in the regulatory architecture of leader sequences between plants and algae. The heatmap analysis revealed that important regions spanned positions –1 to –280 bp in both plant and algal leader sequences, with particular emphasis on the 3′ terminal region. Plant leader sequences exhibited a more heterogeneous attention pattern characterised by distinct peaks. Group-level saliency analysis also identified multiple key regions, with the highest importance scores concentrated approximately 20 bases upstream of the start codon. Perturbation tests further confirmed the critical sensitivity of the region spanning roughly –1 to –20 bp, extending up to -30 within the leader sequences. Overall, all analyses consistently highlighted the central role of the 3′ terminal portion of the leader sequences, particularly the –1 to –30 bp region, in both groups, albeit with differing sensitivities. This finding aligns with previous studies that have identified the region immediately upstream of the start codon as critical for translation initiation in chloroplasts and cyanobacteria, often due to the presence of Shine-Dalgarno-like motifs or other conserved elements (Hirose and Sugiura, 2004a; Kuroda et al., 2007; Drechsel and Bock, 2011). Consistent with these observations, multiple biochemical, genetic, and structural studies have demonstrated that the 20–30 nt region immediately upstream of the start codon in chloroplast 5′-UTRs is indispensable for translation initiation and transcript stability. Mutational analyses of the *psbA* 5′-UTR in *Chlamydomonas reinhardtii* revealed that RNA secondary structures in this proximal region, together with their associated trans-acting factors, are essential for ribosome association and efficient translation (Bruick and Mayfield, 1998). Comparative and computational studies further showed that chloroplast 5′-UTRs commonly form AU-rich stem-loops adjacent to the start codon, which modulate ribosome binding and initiation even in the absence of canonical SD motifs (Higgs et al., 1999). In vivo RNA chemical probing with DMS-MaPseq has additionally confirmed that these structured domains near the AUG codon modulate translation efficiency (Gawroński et al., 2020). Finally, structural and mechanistic investigations have established that ribosomal recruitment in plastids relies on RNA architecture surrounding the start codon, where the −30 to −1 nt segment serves as a principal docking site for mRNA–ribosome interactions (Webster, 2025). Notably, the sensitivity of this region to mutations was greater in algal leader sequences than in the plant sequences, suggesting that the approximately 30-nt region upstream of the start codon in algae is more vulnerable to small changes that can disrupt its function. Therefore, the functional importance of this region should be carefully considered when engineering algal 5′-UTR sequences.

In this context, another promising approach emerging from the current study is the development of hybrid leader sequences for the algal plastome. This approach involves combining the highly functional, algae-specific –1 to –30 bp region with the remaining distal portions derived from plant-origin heterologous sequences. The current results strongly support this strategy: while the model accurately classified 94.87% of plant sequences using their complete length, nearly all plant sequences (99.63%) were misclassified as algal when using only the partial sequences spanning positions –30 to –300. This indicates that the critical species-specific regulatory information primarily resides within the –1 to –30 bp segment, whereas the more distal regions can be of heterologous origin. Such functional hybrid constructs could effectively mitigate undesirable homologous recombination and reduce negative regulatory feedback (Rasala et al., 2011). Therefore, constructing hybrid leader sequences that combine the essential –1 to –30 bp region from algal sequences with distal regions from plant sequences may represent a promising avenue for algal plastid engineering. For further confirmation, the generated hybrid sequence can be analysed using the current trained model; if it is classified with high accuracy as an algal sequence, it can then be considered for additional bioinformatics analyses and experimental investigations.

The hybrid model also accurately distinguished nearly all leader sequences with and without SD motifs in both plants and algae, underscoring the significant differences between these sequence types. The CNN heatmap analysis revealed that attention was broadly distributed across the entire length of leader sequences lacking SD motifs in algae and plants. However, the intensity of this pattern in different regions of leader sequences differed between the algae and plants. This distinction presents challenges for effectively engineering the entire 5′-UTR for synthetic biology or transgene expression in algae. Furthermore, differences in the architectures of these leader sequences between the two groups raise concerns about the reliable transfer of plant sequences into algae, particularly during the critical phase of translation initiation. Consistent with this, the scientific literature lacks a consensus on how plastid 5′-UTRs lacking canonical SD motifs initiate translation (Hirose and Sugiura, 2004b; Drechsel and Bock, 2011). Conversely, leader sequences harbouring SD motifs exhibited similar attention patterns, limited to approximately 20 nt upstream of the start codon in both algae and plants. These results suggest that engineering sequences harbouring SD motifs may be a more predictable strategy than manipulating sequences lacking them.

Group-level saliency analysis revealed that, while the –1 to –20 bp region in sequences containing SD motifs was critical, several other upstream regions also contributed, albeit to a lesser extent. In contrast, sequences lacking the SD motif showed decreased importance in the proximal 20-nt region and increased significance in more distal upstream regions. This shift likely reflects fundamental differences in the regulatory architecture between SD-containing and non-SD leader sequences, consistent with previous findings that, although SD motifs play a critical role in translation initiation in chloroplasts, alternative mechanisms may also be at play (Hirose and Sugiura, 2004b; Scharff et al., 2017). Perturbation tests further confirmed the critical sensitivity of the region from approximately –1 to –20 bp, extending up to –30 bp. This analysis also highlighted that this region of leader sequences containing SD motifs exhibited higher importance scores than those lacking SD motifs.

These findings can be employed to boost the proposed strategy for generating hybrid leader sequences for algal plastomes. The current results suggest that engineering efforts should prioritise the hybrid leader sequences containing SD motifs. This approach is supported by the observation that SD-containing sequences offer a more predictable and distinct regulatory landscape, reducing reliance on other motifs and enabling more rational design. Additionally, incorporating the specific –30 bp region from algae-specific strains could help overcome translation initiation inhibition, which often poses a major bottleneck (Trösch et al., 2018; Zoschke and Bock, 2018). Therefore, constructing hybrid leader sequences containing SD motifs could not only promote efficient translation initiation but also minimise negative regulatory feedback and reduce unwanted homologous recombination in the plastome. Notably, the T7g10 5′-UTR—the only heterologous 5′-UTR recently validated for foreign protein expression in the *C. reinhardtii* chloroplast—contains a Shine-Dalgarno-like motif (Abbasi-Vineh and Emadpour, 2024). The nucleotide composition, particularly the guanine frequency, in algal leader sequences harbouring SD motifs differs from that in their plant counterparts, suggesting that this region warrants particular attention, especially in algae. A practical experimental direction, for example, would be to construct hybrid leader sequences by fusing heterologous 5′-UTRs of highly expressed plastid genes such as *atpH*, *psbD*, *psbC*, *atpE*, and *psbE* from the *Nicotiana tabacum* plastome with approximately 30 bp upstream regions of the corresponding *C. reinhardtii* sequences. This strategy could provide a rational framework for subsequent functional validation in the model microalga *C. reinhardtii*, enabling the assessment of translational efficiency and expression stability within algal chloroplasts.

This is further supported by observations of previous studies that the putative SD sequences found in the 5′-UTRs of many chloroplast mRNAs show considerable variation in size, nucleotide sequence, and distance from the translation initiation codon (Fargo et al., 1998; Hirose and Sugiura, 2004a, 2004b; Wen et al., 2021). However, the anti-SD sequence near the 3′ end of the 16S rRNA is highly conserved in both nucleotide composition and position (Drechsel and Bock, 2011; Scharff et al., 2017; Wei and Xia, 2019; Weiner et al., 2020). In addition, some specific translational activator proteins (not yet fully characterised) may contribute to the initiation of translation in mRNAs whose 5′-UTRs lack canonical SD motifs immediately upstream of the start codon (Hirose and Sugiura, 2004b; Drechsel and Bock, 2011). This makes engineering such sequences more challenging, suggesting that designing sequences containing canonical SD motifs may be a more predictable and reliable strategy.

Therefore, a key output of this study with potential biotechnological applications is the proposal of two emerging strategies for introducing heterologous leader sequences in algal plastome engineering. First, the use of suitable plant-origin heterologous leader sequences that contain algal-like patterns tailored to specific algal strains (dataset available in **Supplementary Material S2**). Second, the development of hybrid leader sequences that incorporate SD motifs by combining the specific ~30 bp upstream from algae-specific strains with distal regions derived from corresponding plant sequences.

Future work will extend this approach to target specific algal and plant taxa prioritised for chloroplast biotechnology, focussing on the design of heterologous 5′-UTRs customised for particular applications. A recently developed data augmentation approach can facilitate this objective, adapted for limited plastome sequence datasets (Abbasi-Vineh et al., 2025). Subsequent efforts will emphasise experimental validation of candidate heterologous leader sequences in key research and commercial strains. While experimental validation in key research and commercial strains remains essential, there is also a need to select suitable derived leader sequences with algal-like patterns tailored to each algal strain. This requires advanced deep learning frameworks capable of analysing full-length leader sequences despite the limited number of available sequences (approximately 100). Although such a developed model is currently under peer review (unpublished), complementary approaches employing machine learning algorithms on quantitative sequence features—such as GC content and minimum free energy (MFE)—extracted from the presented dataset (**Supplementary Dataset S2**) could provide valuable insights. From an applied standpoint, our findings offer significant implications for synthetic biology and genetic engineering, advancing the rational design of translational regulatory elements to optimise plastid expression systems.

## 4 Conclusion

The current deep learning approach confirmed the distinct regulatory architectures of plant and algal 5′ leader regions, as well as sequences with and without SD motifs in both groups, without relying on prior knowledge of established regulatory motifs, sequence structures, or related annotations. The model also identified the evolutionary conservation of regulatory architectures of the sequences. To our knowledge, this is the first deep learning model to analyse translational regulatory sequences of chloroplast genomes, highlighting the importance of interpretable deep learning in regulatory sequence analysis. By integrating advanced interpretative methods—including attention heatmaps, saliency mapping, and perturbation tests—this approach effectively enabled transparent interpretation of its decision-making process. The results from the hybrid CNN-LSTM-Attention-Residual model also provide practical strategies to overcome limitations in transgene translation. These strategies include identifying functional plant-driven heterologous leader sequences and generating hybrid leader sequences that combine plant and algal regions for algal plastomes. These findings have the potential to advance both fundamental understanding and practical applications in chloroplast genetic engineering.

## 5 Materials and methods

### 5.1 Plant and algae genomes and extraction of corresponding 5′-UTRs

To investigate the 5′-UTR sequences of chloroplast genes, chloroplast genomes of plants and algae with a minimum length of 5 kb (either partial or complete genomes) were obtained from the National Centre for Biotechnology Information (NCBI) database (https://www.ncbi.nlm.nih.gov/; accessed on November 29, 2024). In the current study, the chloroplast 5′-UTR sequences of angiosperms, as represented by plant samples, were selected for analysis and compared with those of chloroplast 5′-UTR sequences from algae. The total number of recorded genome sequences for plants and algae was 57,117 and 2,264, respectively. To extract the 5′-UTR sequences, the 300 bp upstream regions of coding sequences (CDS) from the chloroplast genomes of plants and algae were identified separately as putative 5′-UTRs for each group. These extracted sequences, each with a length of 300 bp, may encompass additional components or motifs characteristic of 5′-UTRs and could also serve as “leader” sequences. Despite this potential variability, this length was chosen to ensure comprehensive coverage of relevant information regarding the 5′-UTR sequences. The extracted leader sequences from plants and algae were filtered separately to remove redundant sequences with complete similarity. After filtering, the number of unique leader sequences identified was 884,037 for plants and 110,417 for algae. These sequences were utilised for various analyses, including as input to a deep learning model.

For further analysis, the extracted leader sequences from each group (plants or algae) were divided into two subgroups. The first subgroup included sequences containing one of the consensus Shine-Dalgarno (SD) sequences or sequences derived from them within 20 bp upstream of the start codon (within the -20 bp region of the 5′-UTRs). The identified SD or SD-like sequences included “AGGAGG”, “GAAGGAG”, “AAGGAG”, “AGGAG”, “AAGGA”, “AGGAAG”, “AGGA”, “GGAGG”, “GAAG”, “GGAG”, “GAAG”, “GAGG”, “GGA”, and “AGG”. The second subgroup comprised sequences that lacked any of these motifs within the -20 bp region of the 5′-UTRs.

### 5.2 Implementation of CNN-LSTM hybrid model with attention mechanisms

The proposed deep learning model integrated CNNs for spatial feature extraction, LSTM networks for capturing long-range dependencies, and attention mechanisms for enhanced sequence context modelling. This hybrid architecture was designed to effectively classify nucleotide sequences by leveraging both local and global sequence patterns. Advanced training strategies, including cross-entropy loss, learning rate scheduling, stratified k-fold cross-validation, and early stopping, were employed to optimise model performance and ensure generalisability. The model was implemented using the PyTorch framework (Imambi et al., 2021; Testas, 2024), with mixed precision training to accelerate computation and reduce memory usage.

#### 5.2.1 Data preprocessing and encoding

The leader sequences were stored in separate files, with each file corresponding to a specific class. The sequences were then one-hot encoded to convert them into numerical representations suitable for neural network processing. The encoding scheme mapped nucleotides as follows: A: [1, 0, 0, 0, 0], T: [0, 1, 0, 0, 0], C: [0, 0, 1, 0, 0], G: [0, 0, 0, 1, 0], and N: [0, 0, 0, 0, 1] (for unknown or padded nucleotides).

#### 5.2.2 CNN-LSTM-Attention-Residual hybrid model architecture

The developed model was a hybrid architecture integrating CNNs and LSTMs, followed by two fully connected layers. The CNN component consisted of two main layers. The first layer utilised a single convolutional kernel of size 3, followed by batch normalisation (Balestriero and Baraniuk, 2022) and ReLU activation (Schmidt-Hieber, 2020; Szandała, 2021; Bai, 2022). The second layer employed a multi-kernel approach, simultaneously applying convolutional kernels of sizes 3, 5, and 7, each with appropriate padding to maintain sequence length. Following this, a channel-wise attention mechanism was applied to the concatenated feature maps, dynamically highlighting the most informative channels (Han and Lee, 2020; Zhu et al., 2022). The output was batch-normalised and then max-pooled (Yu et al., 2014). Dropout (rate = 0.5) was applied after the convolutional layers to reduce overfitting (Baldi and Sadowski, 2014). The outputs from both convolutional layers were augmented with residual connections, which help maintain stable gradient flow and mitigate the vanishing gradient problem (Szegedy et al., 2017).

The CNN layers act like a “pattern scanner” that automatically detects important local motifs in sequences, such as translation factor binding sites or conserved nucleotide patterns. The residual connections serve as “memory shortcuts” that preserve important information as it passes through the network, ensuring that early detected features are not lost during deeper processing.

The output was thereafter passed to a bidirectional LSTM layer with 256 hidden units per direction. These LSTM layers enabled the model to learn long-range dependencies in both forward and reverse sequence orientations. The LSTM output was further processed by a self-attention mechanism with four attention heads, which assigned weights to different sequence positions to emphasise the most relevant features (Li et al., 2020; Huang et al., 2023). The resulting attention output was averaged along the sequence dimension to form a context vector that summarises the sequence information. This context vector was passed through two fully connected layers with 256 and 512 neurons, respectively. Each fully connected layer was followed by batch normalisation, ReLU activation, and dropout (rate = 0.3) for regularisation. The final layer used a linear projection to output logits for each class, and a SoftMax activation is applied to yield class probabilities.

The bidirectional LSTM layers function like a “sequence memory” that captures dependencies between distant nucleotides, which is important in biology because regulatory elements or structural features can depend on interactions between far-apart sequence regions. The attention mechanism works like a “spotlight,” focussing the model’s analysis on the most biologically relevant sequence positions. For further details on the biological interpretation of the CNN-LSTM-Attention-Residual model, see **Supplementary Material 1** (**Methods S1**).

#### 5.2.3 Attention heatmap analysis

Model attention patterns were analysed using a two-stage approach to identify sequence regions critical for classification. For a given input sequence *x* of length L, feature maps were extracted from the convolutional CNN and LSTM layers of the trained model.

##### 5.2.3.1 CNN attention weights

The activation maps from the final convolutional layer were averaged across channels to produce a spatial attention score $A^{CNN}$ for each position *i*:


$$A_{i}^{CNN}=\frac{1}{C}\sum_{C=1}^{C}\left|F_{i,\;\;c}\right|$$

Where F ∈ $R^{\left(L\times \;C\right)}$ represented the feature maps with *C* channels, and absolute values were used to quantify activation magnitude.

##### 5.2.3.2 LSTM attention weights

Self-attention weights *α* from the LSTM layer were computed via scaled dot-product attention:


$$\alpha_{\;\mathrm{i,j}}=\;softmax\;(\frac{QK^{T}}{\sqrt{d_{k}}}),\;Q,K=\;LSTM(x)$$

where Q and K were query and key matrices, and $d_{k}$ is the dimension of the key vectors. Position-wise importance was derived by averaging attention scores across all sequence positions:


$$A_{i}^{LSTM}=\frac{1}{L}\sum_{J=1}^{L}\alpha_{\;\mathrm{i,j}}$$

##### 5.2.3.3 Group-level aggregation

Attention weights were aggregated by class to identify consensus patterns. For each class *k* with $N_{k}$ samples, the mean attention heatmap $H^{k}$ was computed as:


$$H_{i}^{k}=\frac{1}{N_{k}}\sum_{n=1}^{N_{k}}{\text{Ã}}_{i}^{\left(n\right)},\;{\text{Ã}}_{i}=\;interpolate\;(A_{i},\;L)$$

Where ${Ã}_{i}$ denoted linearly interpolated attention weights rescaled to the original sequence length (*L* = 300 nt).

#### 5.2.4 Loss function and optimisation

The model utilised cross-entropy loss with Adam optimisation (learning rate = 0.001), incorporating L2 regularisation (weight decay = 1e-5). While the code structure supported advanced training techniques, specific implementation details of learning rate scheduling and gradient clipping required explicit definition in the training loop. The current architecture included dropout layers (0.3-0.5 probability) and batch normalisation between layers as primary regularisation measures.

#### 5.2.5 Training and validation

The model was trained for up to 50 epochs with a batch size of 64. During training, the model’s performance was monitored using a validation set derived from the training data via stratified k-fold cross-validation. The dataset was split into 80% for training and 20% for testing, with stratification to maintain class balance across splits.

#### 5.2.6 K-fold cross-validation for generalisation

To ensure the model’s generalisability and robustness, a 5-fold stratified cross-validation approach was implemented. The training data (80% of the total dataset) was divided into five folds, ensuring that each fold maintained the same class distribution as the original dataset. The model was trained and validated 5 times, with each fold serving as the validation set once, while the remaining four folds were used for training. This process enabled a comprehensive evaluation of the model’s performance across different subsets of the data, thereby reducing the risk of overfitting and providing a more reliable estimate of its generalisation ability.

### 5.3 Evaluation and performance metrics

The performance of the CNN-LSTM model was rigorously evaluated using a comprehensive suite of metrics, including weighted precision, recall, F1-score, ROC-AUC, Matthews Correlation Coefficient (MCC), and precision-recall curves. These metrics were calculated for each fold during cross-validation and averaged to ensure a robust and reliable assessment of the model’s generalisation capability. For final evaluation, the model was tested on a held-out test set comprising 20% of the data, with results reported using the same metrics to facilitate direct comparison with validation performance. Additionally, a confusion matrix was employed to visualise the classification accuracy across different classes, providing detailed insight into the model’s predictive strengths and potential misclassifications.

### 5.4 Post-processing analyses

To further investigate the model’s predictions, several post-processing analyses were performed. These included generating attention maps to visualise the contributions of distinct sequence regions, constructing group saliency maps, and performing perturbation analysis. These approaches enhanced the interpretability of the results, facilitating biological validation and increasing confidence in the model’s applicability for downstream tasks. Detailed descriptions of the group saliency map and perturbation analysis were provided below.

#### 5.4.1 Group saliency map analysis

Group saliency map analysis was performed to identify class-specific sequence regions that most strongly influence the model’s predictions. For each input sequence in the test set, a saliency map was computed by calculating the absolute gradient of the model’s output for the predicted class for each input nucleotide position, as follows:


$$S_{i}=\;|\frac{\partial f(x)}{\partial xi}|$$

where $S_{i}$ denoted the saliency at position *i*, f(x) is the model’s output score for the predicted class, and *xi* is the input at position *i*. These individual saliency maps were grouped according to class labels, summed across nucleotide channels, and averaged across all sequences within each class to yield a group-level saliency profile:


$${\hat{S}}_{i}^{\left(c\right)}=\frac{1}{N_{c}}\sum_{n=1}^{N_{c}}\sum_{i=0}^{n}S_{i,k}^{\left(n\right)}$$

where ${\hat{S}}_{i}^{\left(c\right)}$ was the average saliency at position *i* for class *c*, $N_{c}$ was the number of sequences in class *c*, and *k* indexes nucleotide channels. Peaks in these group-level saliency maps indicated conserved or highly informative regions for each class.

#### 5.4.2 Perturbation analysis

A systematic perturbation analysis was performed to quantify the importance of sequence regions for model predictions. The input sequences were mutated using a vectorised operation that replaced nucleotide segments at specific positions with randomly selected bases. For a sequence *x* of length *L* and a mutation window of size *k* (k=30 nt) starting at position *i*, the mutation operator is defined as:


$$X{'}_{i:i+k}=r\;,\;where\;r\;∼Uniform\;\{A,T,C,G\}$$

The sensitivity score $S_{i}$ for position *i* was computed as the magnitude of the prediction change based on Equation below.

where *f(x)* represents the model’s output logits for the true class, empirically, this was approximated by measuring the difference in prediction accuracy before and after mutation:


$$I_{i}=\frac{1}{N}\sum_{n=1}^{n}[1(y_{n}={\hat{\text{y}}}_{n})-1(y_{n}=\;{\hat{\text{y}}}_{n}^{'})]$$

Here, *N* was the number of samples, $y_{n}$​ was the true label ​ in the original prediction, and ${\hat{\text{y}}}_{n\;}^{'}$ was the prediction after mutation. The analysis was conducted hierarchically: (1) a coarse scan (step size = 3 positions) identified candidate regions, followed by (2) single-nucleotide-resolution probing of regions with $I_{i}$ > 0.1. The results were smoothed using a moving average (window = *L*/10) to highlight biologically plausible features.

The most prominent regions were further analysed using sequence logos, which visualised nucleotide composition and conservation, thereby facilitating biological interpretation of the model’s learned features.

### 5.5 Computational resources for model development and analysis

The integrated CNN-LSTM-Attention-Residual architecture and supplementary analyses were implemented and executed on the Kaggle platform using the CPU-only environment. This environment provides 4 CPU cores and approximately 29 GB of system RAM. The CPU resources are sufficient for model training and evaluation, although training times are longer than in GPU-enabled environments. The main Python scripts employed for these operations are publicly available at https://github.com/MAAbbasi-Vineh/Plastid-5UTRs.

## Data Availability

The original contributions presented in the study are included in the article/**Supplementary Material**. Further inquiries can be directed to the corresponding authors.

## Supplementary material

Methods S1 Biological interpretation of the components of the CNN-LSTM-Attention-Residual model architecture

Supplementary Data Sheet 1 Misclassified plant sequences as algae sequences by the CNN-LSTM-Attention-Residual model

Supplementary Data Sheet 2 Misclassified algae sequences as plant sequences by the CNN-LSTM-Attention-Residual model

## Glossary

Artificial Intelligence: (AI) The simulation of human intelligence by machines to perform tasks like learning and problem-solving
Machine Learning: (ML) A branch of AI where models learn patterns from data to make predictions or decisions without explicit programming
Deep Learning (DL): A subset of ML using multi-layer neural networks to learn complex features from raw datasets automatically
CNN: (Convolutional Neural Network) A deep learning model designed to process data with spatial structure, like images, using convolutional layers
LSTM: (Long Short-Term Memory) A type of recurrent neural network that can remember information over long sequences, applicable for time-series or language data
Attention Mechanisms: Techniques that let models focus on the most relevant parts of input data to improve understanding and predictions
Residual Connections: Shortcut links in neural networks help preserve information and enable the training of very deep models
CNN-LSTM-Attention-: Residual Hybrid Model A combined model leveraging CNNs for spatial features, LSTMs for sequences, attention for focus, and residual connections for better training
Training: The process of adjusting a model’s parameters by exposing it to data so it can learn to make accurate predictions
Validation: Evaluating a trained model on new data (unseen data) to check its accuracy and avoid overfitting
PyTorch framework: An open-source deep learning framework based on Python and the Torch library, used for building and training neural networks
ReLU activation: An activation function introducing non-linearity by outputting zero for negative inputs and the input itself if positive
Recall: A metric measuring the proportion of actual positives correctly identified by the model; also known as sensitivity or true positive rate
F1-score: Harmonic mean of precision and recall, balancing false positives and negatives
ROC: Receiver Operating Characteristic (ROC) curve Plot of true positive rate vs. false positive rate for classifier thresholds
PR: Precision-Recall (PR) curve Shows precision-recall trade-off across classification thresholds, useful for imbalanced data
MCC: Matthews Correlation Coefficient (MCC) Metric for binary classification quality from -1 (worst) to +1 (best)
Heat map attention: A visual tool highlighting parts of input data that a model focuses on, indicating areas with the greatest influence on the output
Saliency map analysis: Visualisation of input areas most influencing model output for interpretability
Perturbation analysis: Method for analysing changes in model output after slight input alterations
Confusion matrix: Table summarising true/false positives and negatives for classification performance
Leader sequence: A sequence located at the 5' end of an mRNA transcript that may include additional components or motifs characteristic of 5′-UTRs
SD: Shine–Dalgarno (SD) sequence A conserved bacterial or plastid mRNA ribosomal binding site that aligns the ribosome for translation initiation
Anti-SD sequence: A sequence complementary to the SD sequence, typically found in the 16S rRNA of the small ribosomal subunit, which base-pairs with the SD sequence to initiate translation
