# Supplementary material for: Deep learning-based investigation of chloroplast translation regulatory sequences
Source: Front Plant Sci. 2025 Dec 11;16:1698951. doi: 10.3389/fpls.2025.1698951 (PMC12738836; doi:10.3389/fpls.2025.1698951)
Supplement: Methods S1 — Biological interpretation of the components of the CNN-LSTM-Attention-Residual model architecture [file DataSheet3.pdf]

Supplementary Methods

Biological interpretation of the components of the CNN-LSTM-Attention-Residual model architecture

Overview

This supplementary file provides a plain-language explanation of the CNN-LSTM-Attention-Residual hybrid model architecture and its components, with biological analogies and examples to aid understanding by molecular geneticists and biologists.

1. Convolutional Neural Networks (CNNs) — Pattern Scanners

CNN layers scan input sequences using small "windows" (kernels) that slide across the sequence, detecting local patterns such as DNA motifs or conserved nucleotide groups. This is similar to how a biologist might scan a DNA sequence for known translation factor binding sites or regulatory elements. For example, a kernel of size 5 looks at every 5-nucleotide segment (like a motif) to find characteristic patterns important for classification.

2. Residual Connections — Memory Shortcuts

Residual connections act like shortcuts that allow information from earlier layers to bypass intermediate layers and be directly passed to deeper layers. This helps prevent the loss of important features during complex processing, ensuring that early detected motifs remain influential in the final decision. It is like a researcher keeping notes from early observations to refer back to later in the study.

3. Long Short-Term Memory (LSTM) Layers — Sequence Memory

LSTM layers are designed to remember information over long distances in sequences. This is biologically relevant because interactions between distant nucleotides or amino acids can influence gene regulation or protein folding. For example, LSTM can capture that a nucleotide at position 10 affects the function of a motif at position 250.

4. Attention Mechanisms — Spotlight on Important Regions

Attention mechanisms assign different weights to sequence positions, allowing the model to focus on the most relevant parts of the sequence when making predictions. It is like a biologist focusing on key regulatory regions or conserved domains when analyzing a gene.

5. Fully Connected Layers — Decision Making

The fully connected layers integrate all extracted features to make the final classification, similar to how a scientist synthesizes all gathered evidence to reach a conclusion.

Summary

This biologized explanation aims to bridge the gap between complex machine learning techniques and biological intuition, making the model more accessible and interpretable for molecular geneticists.

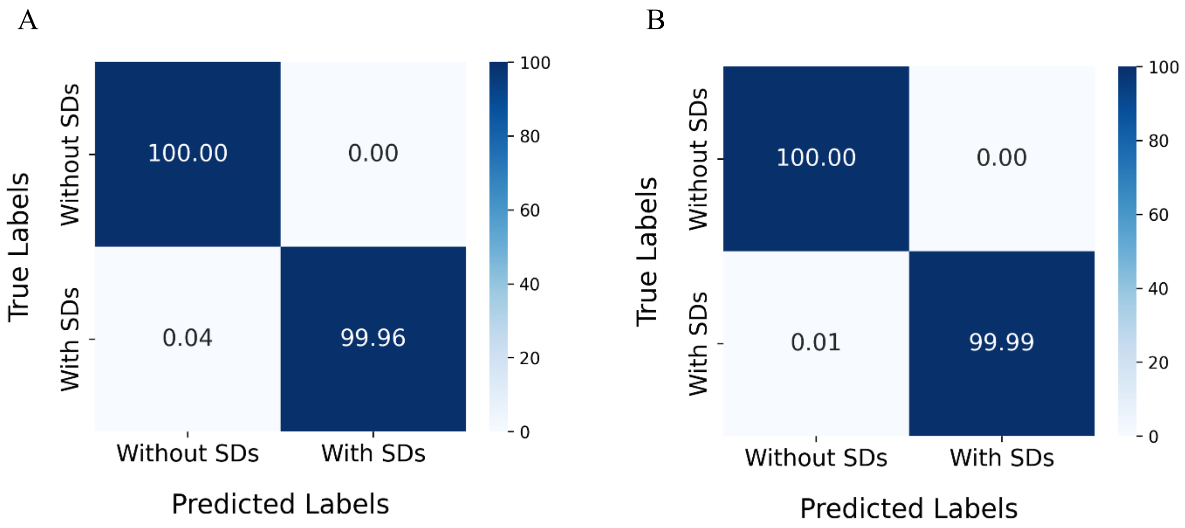

Figure S1. Confusion matrix analysis of leader sequences containing or lacking SD motifs in plants and algae. The matrix evaluates the CNN-LSTM model's binary classification performance for leader sequences with and without SD motifs in (A) plants and (B). Rows denote the true class labels (with SD motifs and without SD motifs), while columns represent the predicted labels. Diagonal elements—top-left for sequences without SD motifs and bottom-right for sequences with SD motifs—reflect the proportion of correctly classified samples. Off-diagonal elements indicate misclassified instances.

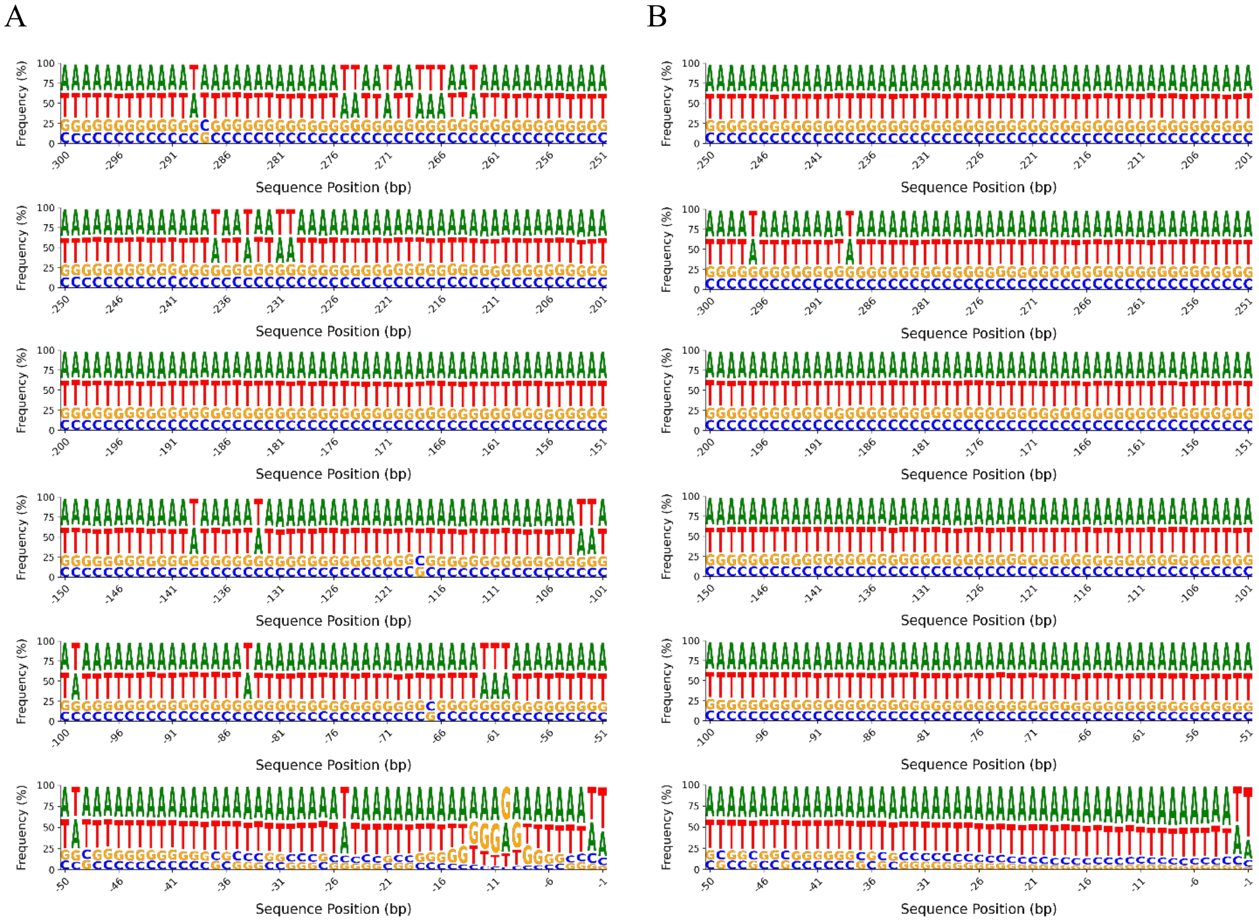

Figure S2. Sequence logos representing the algal leader sequence over the entire 300-nucleotide length, distinguished by the presence or absence of Shine-Dalgarno (SD) motifs. Panels on the left (A) display 5′-UTRs from algae containing SD motifs, while panels on the right (B) show 5′-UTRs lacking SD motifs. For clarity, the full 300-nucleotide region is split into six subplots, each illustrating a 50-nucleotide segment, rather than a single composite plot.

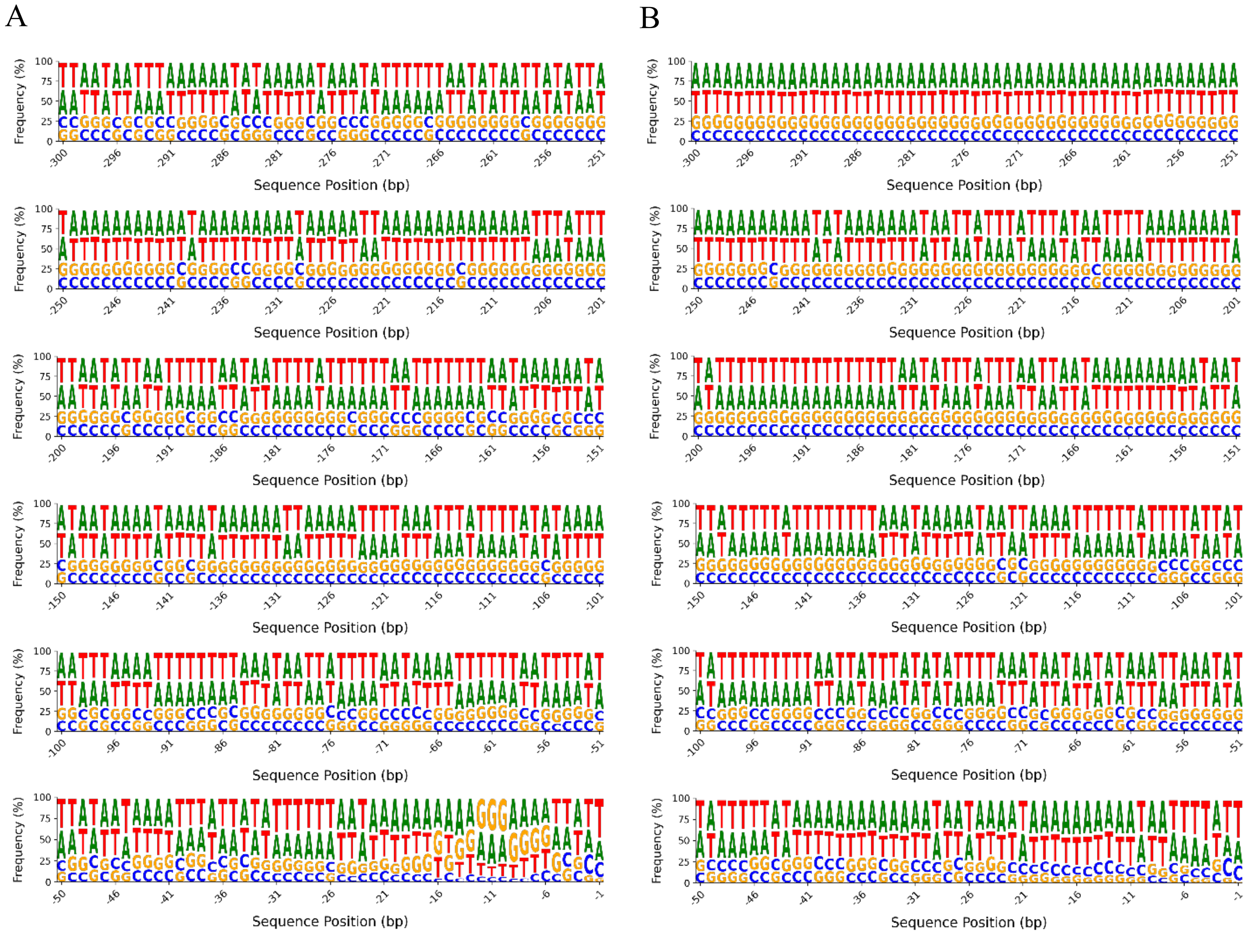

Figure S3. Sequence logos illustrating the full 300-nucleotide length of plant leader sequences were generated based on the presence or absence of Shine-Dalgarno (SD) motifs. The panels on the left (A) represent 5′-UTRs from plants that contain SD motifs, whereas the panels on the right (B) correspond to 5′-UTRs lacking these motifs. To enhance clarity, the entire 300-nucleotide region is divided into six separate subplots, each depicting a 50-nucleotide segment, rather than being displayed as a single combined plot.

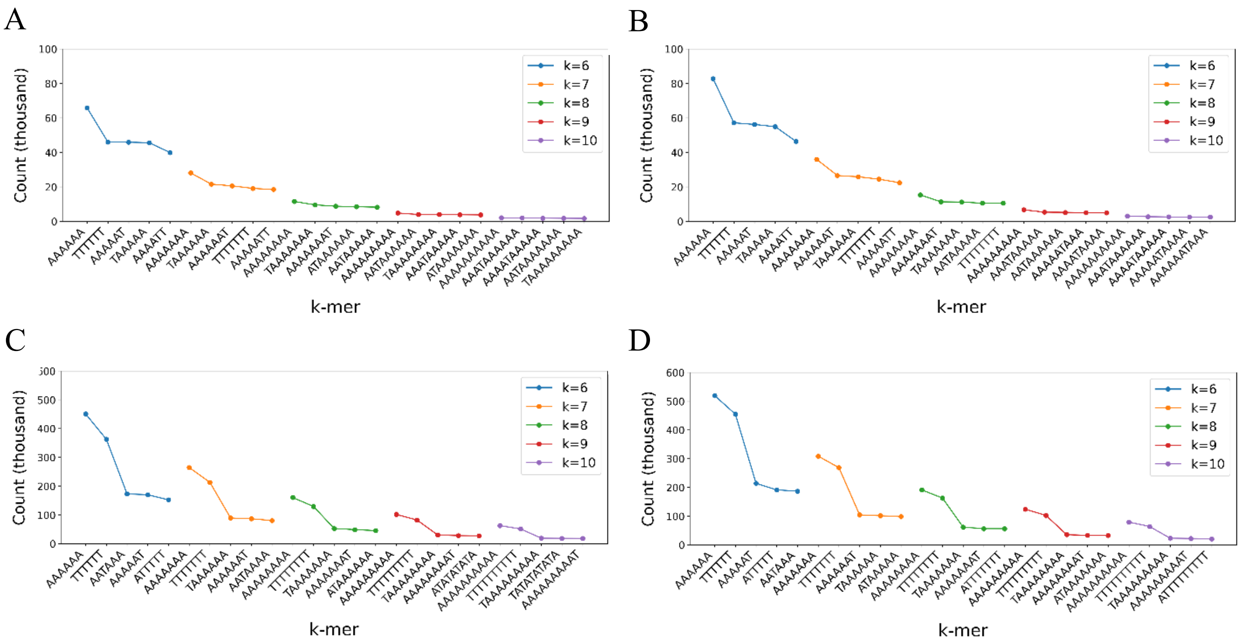

Figure S4. Comparative analysis of top enriched k-mer frequencies across algal and plant sequences with and without Shine-Dalgarno (SD) motifs. Panels (A) and (B) depict the top 5 most abundant k-mers (lengths k=6–12) in algae with and without SD motifs respectively, while panels (C) and (D) show corresponding results for plant sequences. Y-axis values represent occurrence counts in thousands, with line plots connecting k-mers of identical length within each dataset.

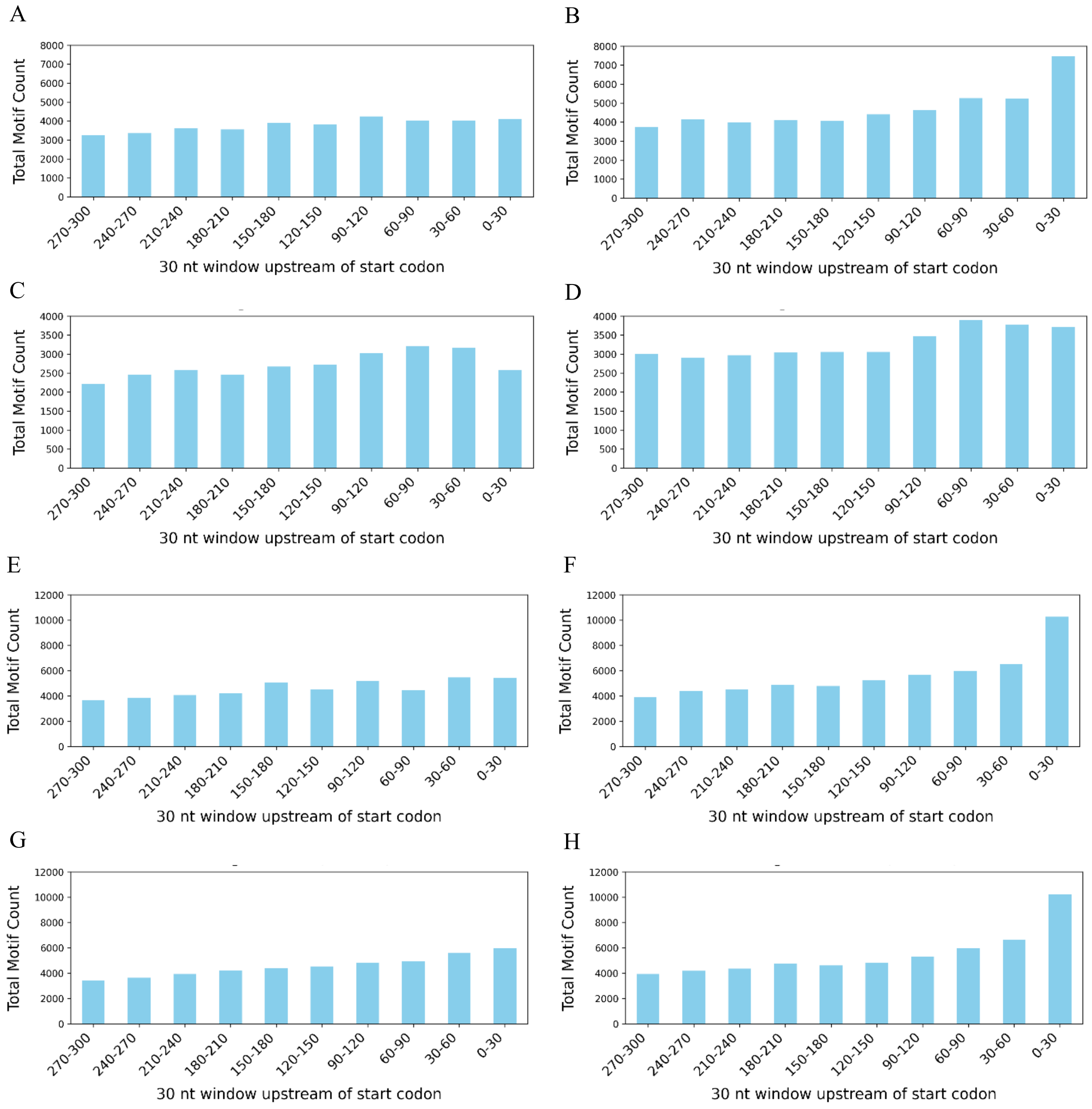

Figure S5. Distribution of a number of high frequent motifs over the entire 300-nucleotide length of Algal leader sequences relative to start codon position. Panels show motif frequency per 30-nt upstream window for: (A-B) "AAAAAA" motif in sequences (A) with and (B) without SD motifs; (C-D) "TTTTTT" motif (C) with and (D) without SD motifs; (E-F) "AAAAAT" motif (E) with and (F) without SD motifs; and (G-H) "TAAAAA" motif (G) with and (H) without SD motifs. Left column (A,C,E,G) represents sequences containing Shine-Dalgarno (SD) motifs; right column (B,D,F,H) shows sequences lacking SD motifs. Window positions (x-axis) indicate distance upstream of start codon (0 corresponds to -1 of start codon). Frequencies represent total motif counts aggregated across all sequences in each dataset.

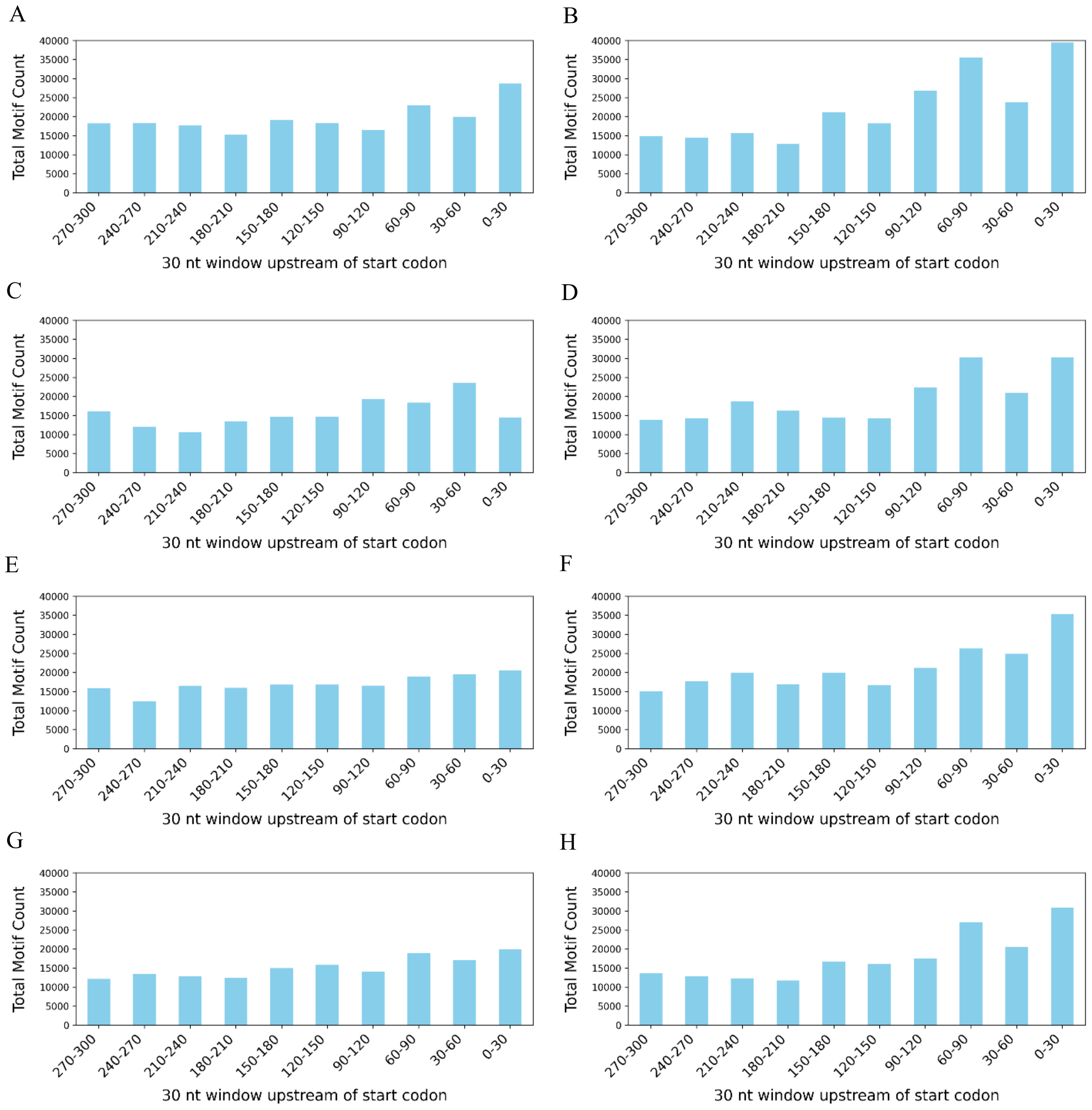

Figure S6. Distribution of a number of high frequent motifs over the entire 300-nucleotide length of plant leader sequences relative to start codon position. Panels show motif frequency per 30-nt upstream window for: (A-B) "AAAAAA" motif in sequences (A) with and (B) without SD motifs; (C-D) "TTTTTT" motif (C) with and (D) without SD motifs; (E-F) "AAAAAT" motif (E) with and (F) without SD motifs; and (G-H) "TAAAAA" motif (G) with and (H) without SD motifs. Left column (A,C,E,G) represents sequences containing Shine-Dalgarno (SD) motifs; right column (B,D,F,H) shows sequences lacking SD motifs. Window positions (x-axis) indicate distance upstream of start codon (0 corresponds to -1 of start codon). Frequencies represent total motif counts aggregated across all sequences in each dataset.

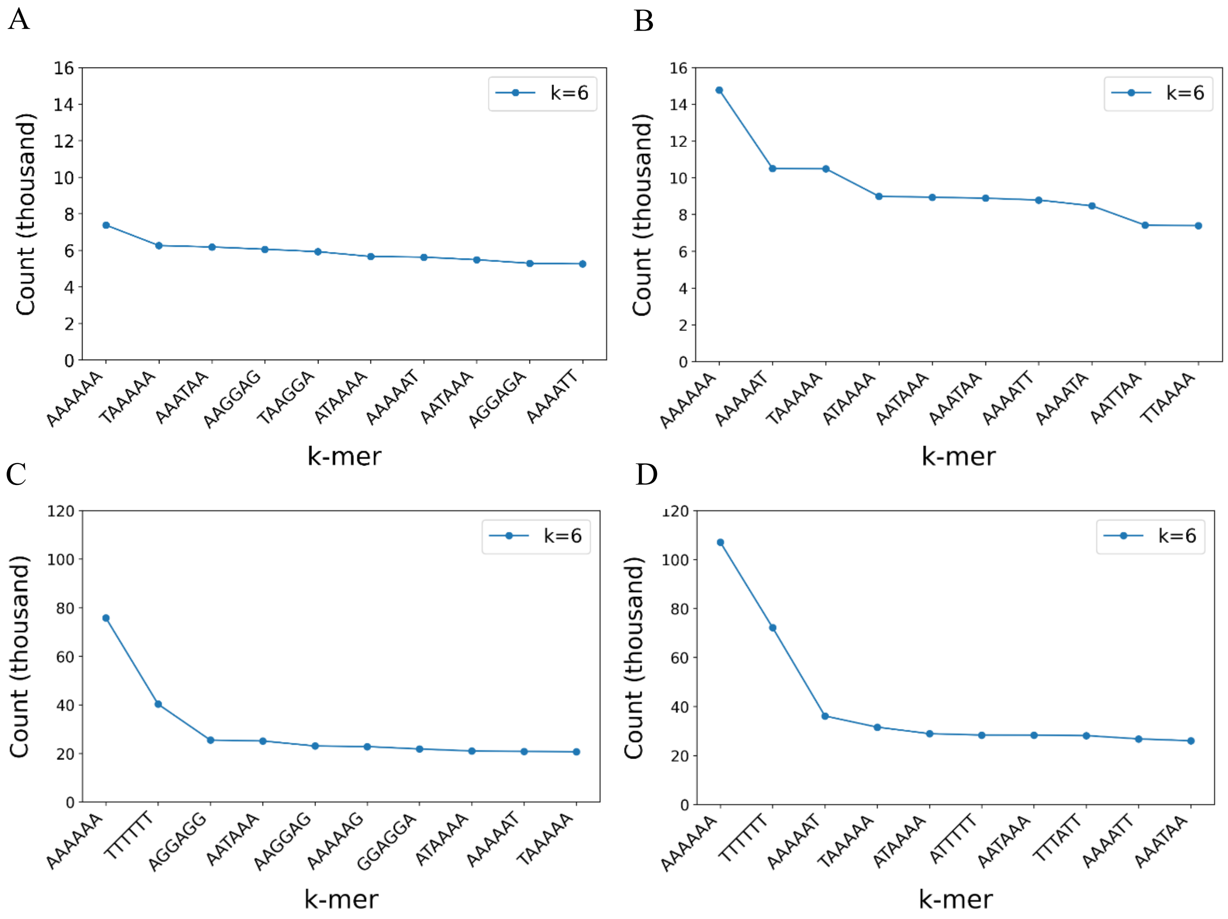

Figure S7. Top enriched 6-mer frequencies in the -1 to -30 region upstream of start codons across algal and plant leader sequences. Panels show occurrence counts (×10³) of the top 10 most abundant 6-mers for: (A) Algae with SD motifs, (B) Algae without SD motifs, (C) Plants with SD motifs, and (D) Plants without SD motifs. Analysis was restricted to the 30-nucleotide window immediately upstream of start codons (positions -1 to -300 in sequence coordinates).

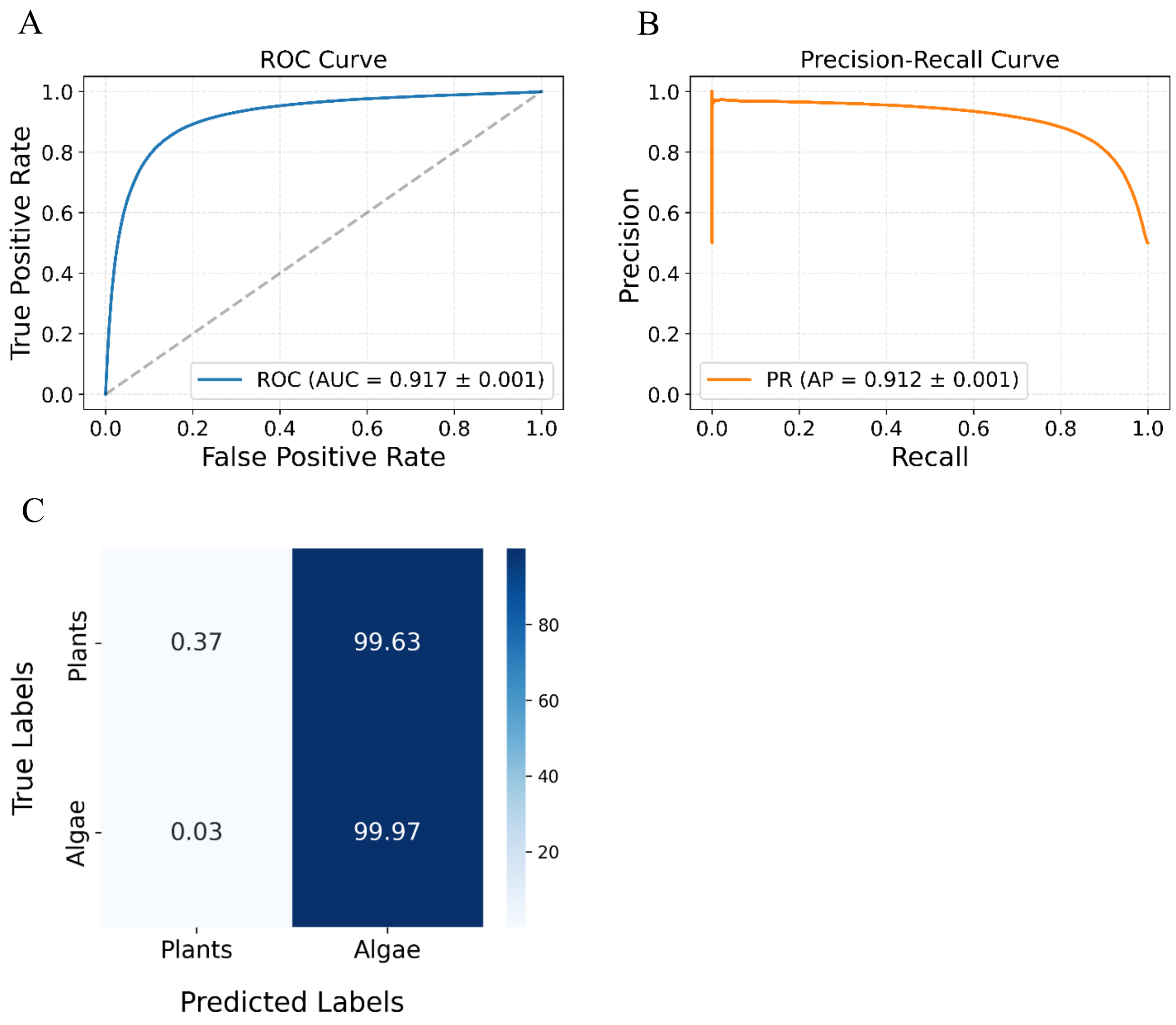

Figure S8. Receiver Operating Characteristic (ROC) and Precision-Recall (PR) curves, along with the confusion matrix analysis, for the classification model on partial leader sequences (270 nt, spanning positions -30 to -300) of plants and algae. Panel (A) displays the ROC curve, where the x-axis represents the false positive rate and the y-axis indicates the true positive rate. Panel (B) shows the PR curve, with recall (sensitivity) on the x-axis and precision on the y-axis. Both curves were generated from the model's predictions on the leader sequence datasets. The average area under the ROC curve (AUC) and the average precision (AP) values, calculated over multiple runs, are annotated on each plot to summarize overall model performance. Panel (C) presents the confusion matrix analysis of the CNN-LSTM model's classification performance. The matrix displays binary classification results, where the rows correspond to the true class labels (Algae and Plant) and the columns indicate the predicted class labels. Diagonal elements (top-left for Plants, bottom-right for Algae) represent the proportions of correctly classified samples, while off-diagonal elements correspond to the percentages of misclassified leader sequences between plant and algae.
